# Genotype-by-Sequencing Analysis of Mutations and Recombination in Pepper Progeny of Gamma-Irradiated Gametophytes

**DOI:** 10.3390/plants10010144

**Published:** 2021-01-12

**Authors:** Yeong Deuk Jo, Han Sol Kang, Hong-Il Choi, Jin-Baek Kim

**Affiliations:** Radiation Breeding Research Team, Advanced Radiation Technology Institute, Korea Atomic Energy Research Institute, Jeongeup 56212, Korea; jyd@kaeri.re.kr (Y.D.J.); ifc1004@nate.com (H.S.K.); hichoi@kaeri.re.kr (H.-I.C.)

**Keywords:** mutation breeding, gamma-rays, gametophyte, recombination, genotype-by-sequencing (GBS)

## Abstract

The irradiation of dry seeds is the most widely-used irradiation method for improving seed-propagated crops; however, the irradiation of other tissues also has useful effects. The irradiation of plant reproductive organs, rather than seeds, for mutation breeding has advantages, such as producing non-chimeric progeny. However, the mutation frequency and spectrum produced using this method have not been analyzed on a genome-wide level. We performed a genotype-by-sequencing analysis to determine the frequencies of single-base substitutions and small (1–2 bp) insertions and deletions in hot pepper (*Capsicum annuum* L.) plants derived from crosses using gamma-irradiated female or male gametophytes. The progeny of irradiated gametophytes showed similar or higher DNA mutation frequencies, which were dependent on the irradiation dose and irradiated tissue, and less biased single base substitutions than progeny of irradiated seeds. These characteristics were expected to be beneficial for development of mutation population with a high frequency of small DNA mutations and performing reverse-genetics-based mutation screening. We also examined the possible use of this irradiation method in manipulating the meiotic recombination frequency; however, no statistically significant increase was detected. Our results provide useful information for further research and breeding using irradiated gametophytes.

## 1. Introduction

Irradiation has long been used in various ways for crop improvement. Mutation breeding, in which mutagenesis by irradiation is used to develop a mutation population, followed by the selection of lines with useful agronomic characteristics, has been most widely used in this area since Stadler [1,2] reported the first induced crop mutation resulting from X-irradiation. In addition to mutation breeding, applications based on the diverse effects of radiation have also been investigated. For example, the inactivation of nuclear genome by irradiation has been used in the introgression of cytoplasmic male sterility (CMS) through protoplast fusions between different species [3] and in the induction of parthenocarpy by pollination with radiation-sterilized pollen [4]. Increasing the meiotic recombination frequency in gametogenesis using irradiation was also attempted based on the assumption that DNA damage, including double-strand breaks caused by irradiation, affects the recombination frequency [5].

In radiation mutation breeding, irradiation conditions, including types of radiation and plant tissues, are factors that affect the spectra and frequencies of DNA mutations, which are important determinants of the range of mutant phenotypes and the size of the mutation population required to obtain a certain proportion of mutant individuals [6]. Research based on the characterization of mutated DNA regions [7,8] or whole-genome sequencing analyses [9,10,11,12] has commonly showed that radiation induces a large number of single-base substitutions (SBSs) and small insertion and deletions (InDels) as well as large deletions and DNA structural variations. The ratio between frequencies of small and large scale mutations have been shown to be closely related to linear energy transfer (LET) of radiation in these research studies. For example, gamma-rays, which are classified as a low-LET radiation and have been most widely used in mutation breeding, induce SBSs predominantly while high-LET radiation such as heavy ion beams and fast neutrons generate higher proportion of large deletions and translocations compared to low-LET radiation [9,10,11,12]. Recently, DNA mutations caused by proton beam irradiation were characterized using a genotype-by-sequencing (GBS) analysis, in which short sequences near DNA regions cut by a specific restriction enzyme are analyzed to investigate SBSs and small InDels in a cost-effective way [13,14]. Regarding irradiated plant tissues, Hase et al. [15] showed, using a whole-genome sequencing analysis, that the irradiation of dry seeds resulted in a significantly higher frequency of DNA mutations, especially InDels, compared with the irradiation of seedlings. However, DNA mutations induced by the irradiation of other plant tissues have not been investigated.

The use of gametophytes irradiated during their developmental stages in mutation breeding has been attempted previously owing to its advantages over seed irradiation. First, progeny obtained from crosses using mutated gametophytes are non-chimeric, unlike chimeric plants generated from irradiated seeds [16]. Second, uninheritable mutations that severely affect the viability of plants can be eliminated at the very early stages of mutation breeding because haploid gametophytes are generally not fertile if they contain critical mutations [17]. Finally, the frequencies and spectra of the mutations are different from those induced by seed irradiation [18]. On the basis of a phenotypic analysis, Kowyama et al. [18] showed that use of pollen obtained from reproductive organs irradiated at specific developmental stages resulted in very high mutation frequencies. Additionally, Viccini and Carvalho [17] reported the possibility of DNA rearrangements after the irradiation of pollen by showing the abnormal behaviors of chromosomes during mitosis in progeny. However, there have been no studies on DNA mutations caused by the irradiation of reproductive organs using a genomics approach. 

In addition to applications in mutation breeding, the irradiation at reproductive organs may be used to manipulate the meiotic recombination frequency. Increasing meiotic recombination leads to an increase in genetic diversity and the introgression of useful characteristics from wild species during breeding process [19,20]. Specific environmental conditions, as well as the genetic engineering of anti-crossover factors, affect the meiotic recombination rate, but the underlying mechanisms remain largely unknown [5,21,22,23]. In Arabidopsis, UV irradiation increased the meiotic recombination frequency by more than threefold [5]. 

In this study, we investigated, using a GBS analysis, the frequencies of mutations, and meiotic recombination events in gametes generated from the irradiated reproductive organs of hot pepper (*Capsicum annuum* L.), to examine the applicability of irradiating these tissues for crop improvement. 

## 2. Results

### 2.1. Development of Pepper Lines by Artificial Crosses Using Irradiated Reproductive Organs, and a GBS Analysis of the Resulting DNA Mutations 

**Figure 1:**
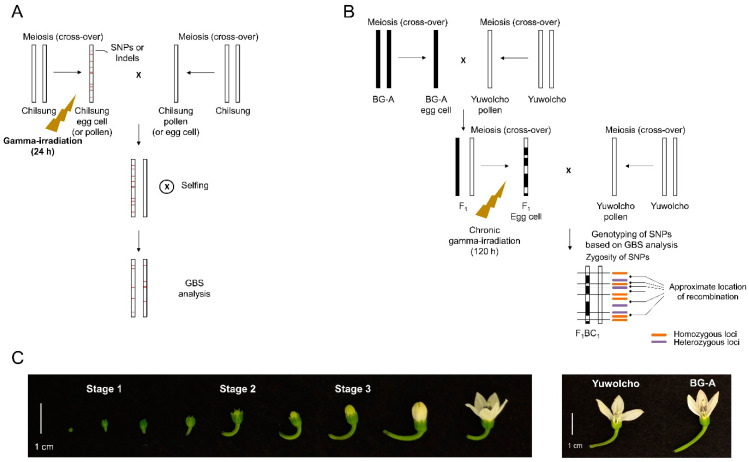
Plant materials and processes for developing mutant lines used for the genotype-by-sequencing (GBS) analysis. (**A**) Crossing and irradiation strategies for the analysis of the mutation frequency and spectrum; (**B**) Crossing and irradiation strategies for the analysis of meiotic recombination; (**C**) Classification of floral buds from ‘Yuwolcho’ based on the developmental stages (left) and phenotypes of flowers from a cytoplasmic male sterility (CMS) line, BG-A.

To investigate the frequencies and spectra of gamma-ray-induced DNA mutations in male or female gametophytes, we gamma-irradiated pepper plants having floral buds at various developmental stages, and then we performed reciprocal crosses with the non-irradiated line (Figure 1A). Crosses in which the irradiated plants were used as seed parents were performed in combinations of three developmental stages of floral buds (based on the time of irradiation) and four irradiation doses (15, 30, 60, and 120 Gy/24 h) (Figure 1C; Table S1). When the irradiated plants were used as pollen parents, pollen from open flowers was used for the cross immediately after irradiation at 15, 30, 60, and 120 Gy. We self-pollinated the plants from seeds obtained from those crosses, and the resulting progeny were used in the GBS analysis. For comparisons with mutant lines developed from irradiated seeds, we gamma-irradiated dry seeds at 30, 60, 120, and 240 Gy, and then we self-pollinated plants from those seeds to develop M_2_ lines. Twofold greater doses were used to treat seeds in comparison to floral buds because the former are more tolerant to irradiation than seedlings [24]. The survival rates of M_1_ seedlings from dry seeds gamma-irradiated at 30, 60, 120, 240 Gy were 89.1, 97.3, 59.5, 16.2%, respectively (Table S2). A GBS analysis was performed using the M_2_ lines for 30, 60, 120 Gy-irradiation since 240 Gy-irradiation was thought to be not applicable for mutation breeding owing to very low survival rate in M_1_ generation.

Crosses using irradiated plants as seed parents showed higher fruit-set rates when floral buds were irradiated at lower doses and later developmental stages were used (Table S1). Irradiation at the highest dose (120 Gy) resulted in the failure of crosses using floral buds from any developmental stage. Crosses using flowers irradiated at the earliest stage (stage 1) did not result in a successful fruit-set in irradiation at most doses, implying that female gametes in the early developmental stage are very sensitive to irradiation. Crosses using pollen from irradiated plants showed fruit-set rates of 40% or higher at any dose, but the fruit obtained after 120-Gy irradiation did not bear any seeds (Table S1). The lines obtained from successful crosses and self-pollination were analyzed using a GBS.

In the GBS analysis, 777 Mb of raw reads were generated per plant (Table 1, Tables S3 and S4). By mapping to the pepper reference genome, trimmed reads were localized to 79,402 regions that were 11.45 Mb and 143.91 bp in total and average length, respectively. These mapped regions corresponded to 0.37% of the reference genome. SBSs and small InDels that were supported by at least five mapped reads (5× sequencing depth) were used to analyze the DNA mutation frequency. 

### 2.2. Frequencies and Spectra of DNA Mutations Induced by the Irradiation of Reproductive Organs

**Figure 2:**
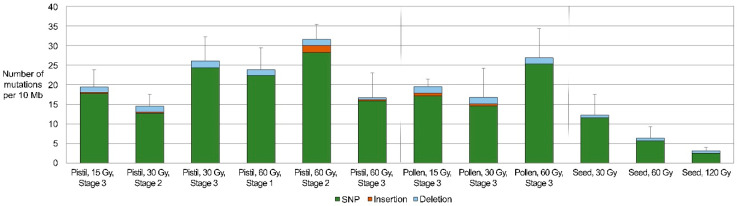
Mutation frequencies in groups of progenies derived from gametophytes irradiated under different conditions. Error bars indicate experimental errors in number of mutations per 10 Mb (*n* = 3).

Using the sequences obtained from the GBS analysis, SBSs and small InDels (1–2 bp) were screened in groups of pepper plants that had been derived from 12 successful cross combinations (Figure 2, Tables S5 and S6). Because the GBS analysis was based on short sequences (Table 1), other structural variations, including large InDels, inversions, and translocations, were not investigated. Although the average densities of the mutations differed depending on the irradiation dose and the irradiated tissue, they were generally similar between cross combinations in which irradiated female (14.5–31.6/10 Mb) and male gametophytes (16.7–26.8/10 Mb) were used. However, these values were similar or higher than those of plants derived from irradiated seeds (3.1–12.3/10 Mb). The frequencies of mutations in female and male gametophytes from reproductive organs irradiated at 60 and 15 Gy, respectively, were significantly higher than those in the seeds irradiated at 60 and 120 Gy, respectively (Figure 2, Table S7). The average of frequencies was also higher in other groups in which irradiated female or male gametophytes were used than in irradiated seeds; however, the differences were not statistically significant (Table S7).

**Figure 3:**
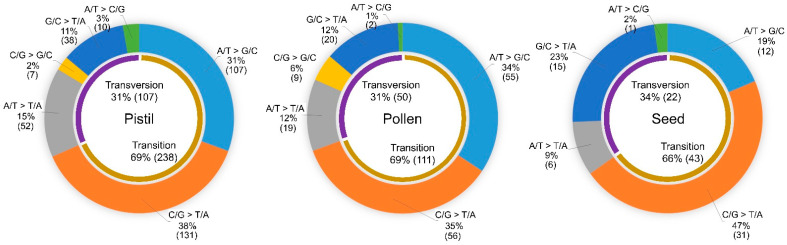
Comparison of single-base substitution (SBS) compositions between progeny from crosses using irradiated pistils, pollen, and seeds. The number of SBSs was indicated in parenthesis.

In all the groups, SBSs accounted for much higher proportions of polymorphisms (81.0–95.3%) compared with small InDels. An analysis of the SBS spectrum showed that the proportions of transitions (31–34%) and transversions (66–69%) were not greatly different between theses irradiated tissues (Figure 3). However, notable differences between reproductive organs and seeds were detected when a detailed analysis was performed. The proportion of transitions from A/T to G/C was higher in reproductive organs (pistil 31% and pollen 34%) than in seeds (19%) (*p*-value < 0.05 in chi-square test). Additionally, the proportion of transversions from G/C to T/A was lower in reproductive organs (pistil 11% and pollen 12%) compared with seeds (23%) (*p*-value < 0.05 in chi-square test). Therefore, the type of irradiated tissue may affect the SBS spectrum.

A methylation sensitive-restriction enzyme (*Ape*KI) was used for the GBS analysis, therefore sequences from genic regions may be enriched in this analysis, limiting the precise determination of the ratio between mutations in genic and intergenic regions. However, a relative comparison between experimental groups was performed (Figure S1), and while the proportion of mutations in exon sequences varied between groups (28.3–61.9%), there was no consistent pattern associated with tissue or irradiation dose.

### 2.3. Development of Progeny Lines by Artificial Crosses Using Irradiated Reproductive Organs, and a GBS Analysis of the Resulting Meiotic Recombination Frequencies

To investigate whether the frequency of meiotic recombination can be manipulated by irradiation, a pepper landrace, Yuwolcho, was crossed with an inbred line, BG-A, which is male sterile owing to maternally-inherited CMS cytoplasm (Figure 1B). The resultant F_1_ plants were irradiated at four different doses (15, 30, 60, and 120 Gy/120 h) when they bore floral buds in various developmental stages (Figure 1C). After backcrosses with Yuwolcho, a GBS analysis was performed using progeny plants to detect the recombination between single nucleotide polymorphism (SNP) markers (Figure 1B). In this strategy, self-pollination, which hampers the development of recombinants, is avoided, and floral organs are irradiated during meiosis to generate female gametes. To meet the first requirement, a CMS line was used as a parent to completely prevent self-pollination both in crosses between parental lines and in backcrosses. To meet the second requirement, chronic irradiation for 120 h performed to cover the whole period during which meiosis occurred. Using a previous cytological analysis in which flower developmental stages of pepper were investigated on the basis of the size and morphology of floral buds [25], stage 1 (Figure 1B) was determined as the period when megagametogenesis starts. After a 5-d irradiation of floral buds that had been in stage 1 when irradiation began, they had proceeded to the later developmental stages that contain functional megaspores [25]. Therefore, we assumed that irradiation of stage 1 floral buds covered the meiotic period. Furthermore, irradiation at stages 0 and 2 corresponded to irradiation before and after meiosis, respectively (Figure 1C)

The GBS analysis was performed for cross combinations from which final progeny could be obtained. The average total length of the raw sequencing reads was 563 Mb (Table 1). The trimmed reads were mapped to 90,051 regions of the pepper genome, covering 0.49% (15.0 Mb) of the reference genome. 

### 2.4. Frequency of Meiotic Recombiantion in the Irradiated Reproductive Organs

**Figure 4:**
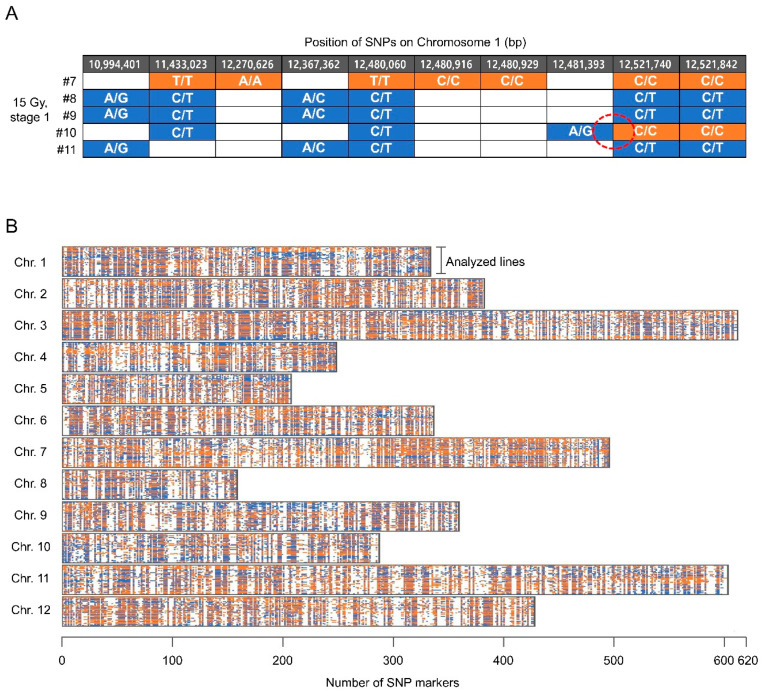
Estimation of recombination using single nucleotide polymorphism (SNP) markers distributed along pepper chromosomes. (**A**) Example of recombination detected based on changes in the zygosity levels between adjacent SNP markers; (**B**) Distributions of SNP markers along the 12 pepper chromosomes.

High quality SNPs that were correctly genotyped in parental and F_1_ lines, and that had sequencing depths that were at least 10×, were selected. Among them, SNP markers that had at least 5× sequencing depths in each progeny line were selected for analysis from each line. As a result, genotypes were obtained from 1554 SNP markers per line on average (Figure 4, Table 2). The average number of markers per chromosome and the average distance between markers in each line were 129.5 and 1.87 Mb, respectively. In total, 55.8% and 44.2% of the SNP markers were determined to be homozygous and heterozygous, respectively. We assumed that recombination occurred between SNP markers when the zygosity levels of adjacent markers differed (Figure 4A). If only a SNP marker was surrounded by two adjacent markers with different zygosity levels, then the marker was excluded from the analysis to reduce false-positive errors. The frequency of recombination per chromosome was determined based on the genotypes of markers dispersed along each chromosome (Figure 4B).

**Figure 5:**
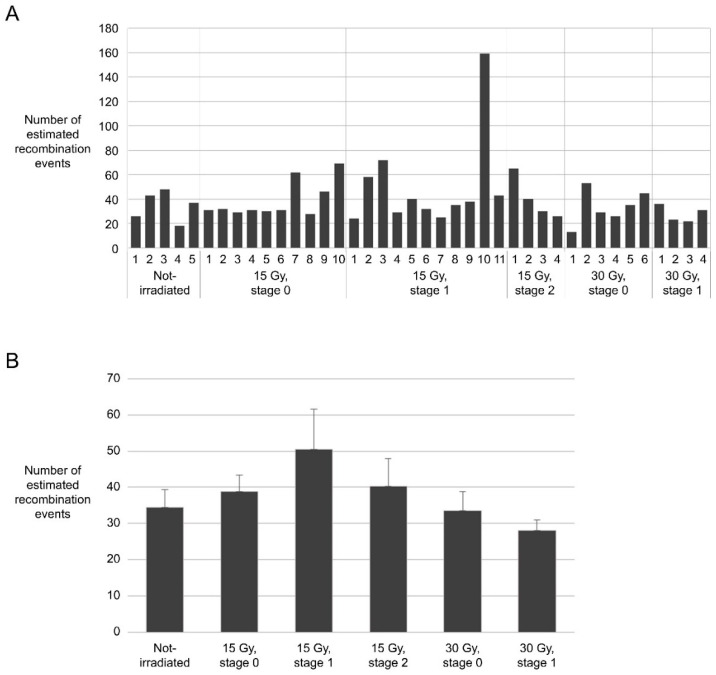
Estimated frequencies of recombination in progeny derived from gametophytes irradiated under different conditions. (**A**) Estimated frequency of recombination in each mutant line; (**B**) Average frequency of recombination estimated in progeny grouped on the basis of irradiation conditions.

The number of recombination events determined by this procedure ranged from 17 to 159, depending on the line (Figure 5A). Although the average number of recombination events was the highest in a group in which stage 1 floral buds were irradiated at 15 Gy (50.3 recombination events per plant on average), the difference from the non-irradiated group (34.4 recombination events per plant on average) was not statistically significant because several lines had very high recombination frequencies (lines 2, 3, and 10) (Figure 5B).

## 3. Discussion

Understanding the frequency and spectrum of mutations resulting from a certain irradiation condition is very important for designing an efficient strategy, and determining an adequate population size, for mutation breeding. Although various irradiation conditions, such as the type of radiation, irradiation dose, dose rate, target tissue, and sample pretreatment, have been applied in mutation breeding, sequencing analyses to obtain genome-scale views of DNA mutations have only been performed recently [9,10,11,12,13,14,15]. We first carried out a genomic analysis of DNA mutations and recombination events in gamma-irradiated reproductive organs to examine the feasibility of using them for crop improvement. Because research that surveyed various irradiation conditions in reproductive organs is limited, we analyzed many groups of plant samples subjected to different irradiation conditions rather than focusing on samples subjected to a specific condition. Therefore, the GBS analysis method, which is cost-effective and suitable for genome-wide analyses of small-scale sequence changes using sequences near endonuclease restriction sites, was applied to analyze a large number of plant samples instead of the whole-genome sequencing method for a small number of samples. 

The frequencies of DNA mutations in progeny of cross combinations using reproductive tissues were similar, or higher, compared with those (M_2_ lines) derived from irradiated seeds. Shirasawa et al. [26] showed that the frequency of SBSs in M_3_ lines derived from gamma-irradiated tomato (Micro-Tom) seeds was 1.25 SBS/10 Mb on average, which was estimated to be 1.5 SBS/10 Mb in the M_2_ generation, if it is assumed that a quarter of the heterozygous mutation are lost through Mendelian segregation in the following M_3_ generation. The frequencies of SBSs in pepper M_2_ lines derived from irradiated seeds were comparable or higher (2.5–11.5 SBS/10 Mb) than the estimated value for tomato. Therefore, considering that the tomato sequencing was performed using lines in a mutation population designed for functional genomics studies and that SBSs accounted for the largest proportion of mutations (77.8%) in that analysis [26,27], we expected that the mutation frequency in progeny derived from the irradiation of pepper floral organs to be similar to, or higher than, that required for the development of a practical mutation population. 

The irradiation of female and male gametophytes commonly showed different spectra of induced SBSs from those detected from irradiated seeds. The substitution of C/G to T/A, results in an extremely high proportion of spontaneous and ethyl methanesulfonate-induced mutations [26,28], and these substitutions accounted for approximately half of SBSs in plants derived from irradiated seeds in our study. However, in progeny from irradiated female and male gametophytes, the proportions of A/T to G/C (31% and 34%, respectively) were similar to C/G to T/A (38% and 35%, respectively), and were significantly higher than that of progeny from irradiated seeds (19%). Among transversion mutations, G/C to T/A was the major type of substitution in irradiated seeds, while the proportions of G/C to T/A and A/T to T/A substitutions were similar in progeny derived from irradiated reproductive tissues. Overall, the SBS composition in reproductive tissues was relatively even; therefore, less bias in the amino acid changes was expected in mutated genes as shown in mutagenesis using fast neutrons [10]. Differences in SBS spectra were also reported in comparisons between irradiated seedlings and seeds [15]. The SBS composition in seedlings was greatly different from that in the reproductive tissues analyzed here. Therefore, differences in physiological characteristics or gene expression patterns between tissues appear to affect the SBS spectrum.

The well-known advantages of generating progeny composed by uniform cells (not chimeric) in the next generation (M_2_) and a relatively high SBS frequency when using mutated gametophytes, allow the employment of a reverse genetics-based approach to mutation breeding based on mutated gametophytes rather than irradiated seeds. Reverse-genetics approaches using induced local lesions in genomes (TILLING) approach or next-generation sequencing [29] may be attempted in M_2_ generations only after the cultivation of large M_1_ populations when seeds irradiated or treated with chemicals are used because the M_1_ individuals are chimeric. The use of irradiated gametophytes may reduce the time and labor required to cultivate large numbers of plants to obtain M_2_ seeds because a large number of non-chimeric M_2_ seeds are obtained from a few crosses using irradiated reproductive organs of crops that bear many seeds in a fruit (e.g., pepper). The resulting M_2_ lines carry all heterozygous mutations that can be detected by reverse-genetics approaches, although mutant phenotypes from recessive mutant alleles cannot be investigated in this generation.

The limitation of GBS is the inability to screen DNA structural variations and InDels longer than 2 bp. Viccini and Carvalho [17] predicted the presence of large DNA rearrangements in progeny derived from irradiated pollen. However, we hypothesized that the frequency of large structural variations would be relatively low in progeny from mutated gametophytes because a substantial proportion of the large structural variations affect the viability of gametes, and therefore, they cannot be inherited by the next generation [30]. In addition, SBSs were shown to be the predominant type of mutation induced by gamma-rays [12,26]. Further research based on whole-genome sequencing may reveal the frequencies and characteristics of these types of mutations and this will enable the estimation of the mutated gene frequency that is required for the determination of the mutation population size.

We speculated that DNA damage caused by irradiation may increase recombination during meiosis; however, a significant change in the recombination frequency was not detected. Thus, inducing double-strand breaks is not enough to increase the recombination frequency. Crossover formation during meiosis is strictly controlled by regulatory proteins; therefore, engineering the genes encoding those proteins is effective for manipulating the recombination frequency [19]. Furthermore, environmental factors, such as temperature and nutrition, have also been shown to affect the recombination frequency [21,22,23]. Preuss and Copenhaver demonstrated that UV-irradiation increases the recombination frequency in Arabidopsis [5]. Considering our results, this might reflect the effects of UV-irradiation on controlling recombination (e.g., alteration gene expression profiles) rather than UV-irradiation causing DNA damage. Although the average frequency was not significantly changed, we detected large numbers of recombination events in a few lines in the group derived from irradiation during meiosis (e.g., line number 10 irradiated at 15 Gy). Thus, irradiation during a very specific developmental stage may be effective in inducing recombination. Kawyama et al. [18] showed that a difference of only 1 day in the developmental stage at which male gametophytes were irradiated greatly affects the mutation frequency in mutation breeding using pollen, which indicated that the developmental stage is very critical for successful irradiation. An analysis of groups classified on the basis of subdivided developmental stages is required to examine this possibility.

In conclusion, we characterized mutations and recombination events in reproductive organs of gamma-irradiated pepper plants to examine the feasibility of using irradiated gametophytes for crop improvement. Progeny of irradiated gametophytes showed similar or higher mutation frequencies and SBS compositions compared with those from irradiated seeds, indicating that efficient mutation breeding using irradiated gametophytes is possible. However, the recombination frequency was not significantly changed in progeny from irradiated gametophytes, showing that irradiation during meiosis may not be applicable for the manipulation of the recombination frequency. The information produced from using the genomic approach in this study will aid in designing a strategy for mutation breeding that employs irradiated gametophytes. 

## 4. Materials and Methods

### 4.1. Plant Materials and Gamma-Irradiation

In the analysis of mutation frequency and spectrum, a Korean landrace of hot pepper (*Capsicum annuum* L.), ‘Chilsung’, was used as the plant material. After cultivation in pots (25 × 25 cm) in a greenhouse for 3 months, plants bore floral buds during various developmental stages (Figure 1C) that were classified on the basis of the size and morphology of floral buds, using criteria from previous research [25]. Stage 1 in our study corresponded to bud stage 3 of Sandoval-Oliveros et al. [25]. In this stage, buds are green and 3 mm in diameter and the stigma starts to form. Microscopic analyses showed that megagametogenesis began and male meiosis occurred during this stage [25]. At stage 2, petals grow above sepals and floral buds contain unicellular pollen [25]. Stage 3 is marked by protruding white petals, and floral buds contain bicellular pollen grains [25]. Stages of floral buds were marked with ties just before irradiation. Three plants with floral buds at these stages were gamma-irradiated each at 15, 30, 60, and 120 Gy for 24 h at the Korea Atomic Energy Research Institute (Republic of Korea) using a ^60^Co gamma irradiator. After irradiation, mature pollen from open flowers of irradiated plants was used for crosses with non-irradiated plants (Figure 1A). On the other hand, floral buds at three stages were grown until they reached the stage next to stage 3 (flowers are closed and white petals are longer than calyx) and were used as females in crosses with pollen from non-irradiated plants. The progeny from these cross combinations were self-pollinated and the GBS analyses were performed using the resulting next-generation plants (Figure 1A). For the comparative analysis, dry seeds of Chilsung were gamma-irradiated at 30, 60, 120, and 240 Gy using the same facility. The plants from seeds were self-pollinated, and the resulting M_2_ plants were used for the GBS analysis. 

In the analysis of recombination frequency, a Korean landrace of hot pepper (*C. annuum* L.), ‘Yuwolcho’, and a CMS inbred line, BG-A, that were used as male and female parents to obtain F_1_ plants from this cross were cultivated in pots (25 × 25 cm) in a greenhouse for 3 months to obtain floral buds during diverse developmental stages. The floral buds at stages 1 and 2 were marked as described above before irradiation. Chronic irradiation was performed for three plants each at 15, 30, 60, and 120 for 120 h in a gamma phytotron, which is a facility specialized for long-term gamma-irradiation with ^60^Co gamma irradiator in an environmentally controlled room at the Korea Atomic Energy Research Institute. After irradiation, floral buds at stages 1 and 2 were grown until they reached the stage next to stage 3 (described above) and were used as females in crosses with pollen from non-irradiated Yuwolcho plants (Figure 1B). Additionally, the flowers that reached stage 3 at 1 month after irradiation were used for crosses when they had developed to the next stage. We defined the floral buds in this case as stage 0 because they were not yet floral buds at the time the irradiation was performed. The resultant F_1_BC_1_ plants were used for the GBS analysis. 

### 4.2. GBS Analysis

Genomic DNA was isolated from leaf tissue using a GeneAll Exgene™ Plant SV mini kit (GeneAll, Seoul, Korea). The GBS library preparation using extracted DNA and the sequencing were performed by SEEDERS Inc. (Daejeon, Korea). DNA was digested with the restriction enzyme *Ape*KI and adaptors were ligated. Then, samples were cleaned after pooling, and amplified by PCR. The GBS library prepared in this process was used for next-generation sequencing using the Illumina HiSeq 2500 platform (San Diego, CA, USA). Sequences were demultiplexed based on barcode sequences. Trimming of adapter sequences, low quality sequences, and sequence shorter than the minimum length was performed using the cutadapt (version 1.8.3) program [31], and the DynamicTrim and LengthSort programs in the SolexaQA (v.1.13) package [32], respectively. The minimum phred score in DynamicTrim was 20, and the minimum length in LengthSort was 25 bp. The cleaned reads were mapped to the pepper reference genome sequences [CM334 (*Capsicum annuum* L.) v1.6] from the pepper genome platform (http://peppergenome.snu.ac.kr) using the BWA (0.6.1-r104) program [33]. From the obtained BAM format files, SBSs, InDels, and consensus sequences were detected by comparison with the reference genome using SAMtools (0.1.16) [34]. In this analysis, minimum values of mapping quality for SNPs, mapping quality for InDels, and read depth were 30, 15, and 3, respectively. For the comparative analysis between samples, SBS and InDel matrices integrated among samples were constructed. Finally, the SNP and InDel sequences that were common in non-irradiated samples (original lines) were compared with those of samples from irradiated plants (mutant lines) to extract SNP and InDels polymorphic between original and mutant lines. The minimum depth for polymorphic sequences was 5× in this analysis.

### 4.3. Analysis of Mutation Frequencies and Spectra

SBSs (described as SNPs above) and InDels that were common to the four non-irradiated plants and for which the minimum sequencing depth was 5× in a specific mutant line were selected for analysis. The average frequencies of SBSs and InDels were calculated from three plants per irradiation condition. A statistical analysis was performed to examine the differences between groups using Student’s *t*-test at a *p*-value threshold of 0.05. The positions of SBSs were classified to be on exon, intron, or intergenic region by comparison with the reference genome. SBSs were classified as six categories according to type of substituted nucleotide. For frequency of SBS classified as each category, the statistical significance of differences between three groups of lines classified according to irradiated tissues were examined by chi-squared test using IBM SPSS Statistics v22.0 (IBM, Amonk, NY, USA).

### 4.4. Analysis of Recombination

To minimize false positives, SNPs for which the minimum sequencing depth was 10× in the each pool of parental (four plants for each parental line) and F_1_ (four plants) lines were screened. Then, the SNPs that were identical among reads from each parental line and showed an allele frequency between 25% and 75% in F_1_ lines were selected. Finally, SNPs for which the minimum sequencing depth was 5× and showed an allele frequency of 100% (homozygous SNP) or 25–75% (heterozygous SNP) in reads from each mutant line were selected. Each SNP was localized to the 12 chromosomes of the reference genome. If the zygosity of adjacent SNP markers differed, we assumed that a recombination event had occurred between the markers. However, if only one SNP marker was surrounded by two adjacent markers with different zygosity levels, then the marker was excluded from the analysis to reduce false-positive errors. A statistical analysis was performed to examine the differences between groups classified by irradiation conditions on the basis of Student’s *t*-tests in which the *p*-value threshold was 0.05.

## Figures and Tables

**Table 1:** Statistics for the genotype-by-sequencing (GBS) analysis.

Characteristics of GBS Analysis	Average Among Lines	
	Analysis of Mutation Frequency	Analysis of Recombination Frequency
Number of raw reads	7,701,874	5,575,688
Total length of raw reads (bp)	777,889,296	563,144,465
Number of trimmed reads	6,498,878	4,978,217
Total length of trimmed reads (bp)	498,825,520	417,179,109
Average length of trimmed reads (bp)	76.74	83.92
Number of mapped reads (%)	6,176,211 (95.10)	3,143,631 (68.26)
Number of mapped region	79,402	90,051
Average depth of mapped region	35.27	14.33
Total length of mapped region (bp)	11,449,437	14,965,995
Average length of mapped region (bp)	143.91	165.7
Reference Genome coverage (%)	0.37	0.49

**Table 2:** Characteristics of single nucleotide polymorphism (SNP) markers used for the estimation of recombination frequencies.

Characteristics of SNP Markers	Statistics
Total number of applied SNP markers ^z^	4467
Average number of applied SNP markers (per line)	1554
Range of number of applied markers	1399–1732
Percentage of markers scored to be homozygous (%)	55.78
Percentage of markers scored to be heterozygous (%)	44.24
Average number of applied markers per chromosome	129.50
Average distance between applied markers on reference genome (Mb)	1.87

^z^ These markers include those used for genotyping at least one of the mutant lines.

## Data Availability

Data is contained within the supplementary material. The data presented in this study are available in Tables S3–S6.

## Supplementary Materials

Supplementary Materials can be found at https://www.mdpi.com/2223-7747/10/1/144/s1, Table S1: Results of crosses using irradiated gametophytes, Table S2: Survival rate of seedlings investigated one month past gamma-irradiation, Table S3: Statistics for the genotype-by-sequencing (GBS) analysis performed for analysis of mutation frequency, Table S4: Statistics for the genotype-by-sequencing (GBS) analysis performed for analysis of recombination frequency, Table S5: Information of single base substitutions (SBSs) detected by the genotype-by-sequencing (GBS) analysis performed for analysis of mutation frequency, Table S6: Information of small InDels detected by the genotype-by-sequencing (GBS) analysis performed for analysis of mutation frequency, Table S7: Results of pair-wise t-tests between groups of progeny derived from gametophytes irradiated under different conditions, Figure S1: Frequency of single-base substitutions (SBSs) according to location on genome in groups of progeny derived from gametophytes irradiated under different conditions

## Abbreviations

GBS	Genotype-by-sequencing
SBS	Single-base substitution
InDel	Insertion and deletion
CMS	Cytoplasmic male sterility
SBS	Single-base substitution
SNP	Single nucleotide polymorphism
