# Supplementary material for: Genotype-by-Sequencing Analysis of Mutations and Recombination in Pepper Progeny of Gamma-Irradiated Gametophytes
Source: Plants (Basel). 2021 Jan 12;10(1):144. doi: 10.3390/plants10010144 (PMC7827419; doi:10.3390/plants10010144)
Supplement: Supplementary file 1 [file plants-10-00144-s001.pdf]

Table S1. Results of crosses using irradiated gametophytes

Tissue used for cross in irradiated plants	Dose (Gy)	Floral bud stage	No. cross trials	No. successful crosses [z]	Cross success rate (%)	No. seeds per fruit	Percentage of seeds compared to wild type
Pistil	15	1	10	0	0	-	-
		2	10	1	10	0	0
		3	10	8	80	60.1	88.8
	30	1	8	0	0	-	-
		2	10	1	10	60	88.6
		3	10	6	60	51.3	75.8
	60	1	9	1	11.1	12	17.7
		2	9	1	11.1	72	106.4
		3	10	5	50	60	88.6
	120	1	9	0	0	-	-
		2	9	0	0	-	-
		3	9	0	0	-	-
Pollen	15	3	5	5	100	28.8	42.5
	30	3	5	2	40	14	20.7
	60	3	5	2	40	4.4	6.5
	120	3	5	3	60	0	0

[z] A cross was deemed successful if it produced fruit

Figure S2. Survival rate of seedlings investigated one month past gamma-irradiation

Dose (Gy)	Survival rate (%)
0	100
30	89.2
60	97.3
120	59.5
180	29.7
240	16.2

Table S3. Statistics for the genotype-by-sequencing (GBS) analysis performed for analysis of mutation frequency

Irradiated tissue	Dose (Gy)	Flower stage	Individual ID	Sum of trimmed reads	Total length of trimmed reads (bp)	Average length of trimmed reads (bp)	No. mapped reads	No. mapped region	Median depth of mapped region	Total length of mapped region (bp)	Reference genome coverage (%)
Female gamatophyte	15	3	**F15-3-1**	3,331,488	256,781,467	77.08	3,191,585	79,857	9.56	11,697,657	0.3818%
Female gamatophyte	15	3	**F15-3-2**	3,812,844	294,552,877	77.25	3,646,748	75,514	9.93	10,971,862	0.3581%
Female gamatophyte	15	3	**F15-3-3**	7,555,408	579,597,596	76.71	7,233,120	91,713	11.29	13,683,153	0.4466%
Female gamatophyte	30	2	**F30-2-1**	3,923,878	301,256,756	76.78	3,745,783	80,011	10.00	11,885,966	0.3879%
Female gamatophyte	30	2	**F30-2-2**	5,001,472	385,100,490	77.00	4,785,353	81,840	10.98	12,109,828	0.3952%
Female gamatophyte	30	2	**F30-2-3**	6,101,154	467,579,898	76.64	5,841,826	79,271	11.92	11,105,833	0.3625%
Female gamatophyte	30	3	**F30-3-1**	1,805,870	139,027,873	76.99	1,741,653	51,913	9.54	7,166,473	0.2339%
Female gamatophyte	30	3	**F30-3-2**	6,961,892	527,221,013	75.73	5,659,775	77,185	11.79	10,983,539	0.3585%
Female gamatophyte	30	3	**F30-3-3**	2,729,462	207,256,877	75.93	2,282,204	55,875	10.28	7,692,072	0.2511%
Female gamatophyte	60	1	**F60-1-1**	5,436,460	416,911,103	76.69	5,216,746	78,284	11.40	11,439,845	0.3734%
Female gamatophyte	60	1	**F60-1-2**	2,063,478	158,617,035	76.87	1,970,468	48,077	10.68	6,717,862	0.2193%
Female gamatophyte	60	1	**F60-1-3**	3,135,464	240,760,728	76.79	3,003,281	69,291	9.83	10,080,245	0.3290%
Female gamatophyte	60	2	**F60-2-1**	2,500,234	192,193,723	76.87	2,395,260	54,330	10.92	7,670,971	0.2504%
Female gamatophyte	60	2	**F60-2-2**	1,877,012	144,479,970	76.97	1,794,293	50,302	9.87	6,965,339	0.2273%
Female gamatophyte	60	2	**F60-2-3**	4,376,176	338,116,784	77.26	4,201,186	76,625	10.53	11,186,921	0.3651%

Irradiated tissue	Dose (Gy)	Flower stage	Individual ID	Sum of trimmed reads	Total length of trimmed reads (bp)	Average length of trimmed reads (bp)	No. mapped reads	No. mapped region	Median depth of mapped region	Total length of mapped region (bp)	Reference genome coverage (%)
Female gamatophyte	60	3	**F60-3-1**	7,883,426	607,870,070	77.11	7,566,231	95,582	11.48	14,239,198	0.4647%
Female gamatophyte	60	3	**F60-3-2**	4,288,438	328,453,867	76.59	4,089,232	75,480	10.46	11,029,538	0.3600%
Female gamatophyte	60	3	**F60-3-3**	1,759,278	134,340,739	76.36	1,689,188	51,498	9.18	6,994,731	0.2283%
Male gamatophyte	15	3	**M15-3-1**	3,950,014	303,425,102	76.82	3,792,398	68,936	11.03	9,876,802	0.3224%
Male gamatophyte	15	3	**M15-3-2**	4,056,720	310,111,296	76.44	3,895,940	66,301	11.40	9,527,108	0.3110%
Male gamatophyte	15	3	**M15-3-3**	4,429,264	339,376,596	76.62	4,255,859	76,222	10.95	11,112,140	0.3627%
Male gamatophyte	30	3	**M30-3-1**	7,373,486	563,750,676	76.46	7,081,965	87,982	11.35	13,061,260	0.4263%
Male gamatophyte	30	3	**M30-3-2**	2,036,216	155,764,055	76.50	1,930,690	54,182	9.42	7,482,042	0.2442%
Male gamatophyte	30	3	**M30-3-3**	8,234,872	629,725,374	76.47	7,786,752	88,180	11.90	12,721,833	0.4152%
Male gamatophyte	60	3	**M60-3-1**	2,784,308	212,651,716	76.38	2,659,047	66,681	9.26	9,436,626	0.3080%
Male gamatophyte	60	3	**M60-3-2**	2,787,420	213,359,563	76.54	2,658,920	58,418	10.48	8,288,396	0.2705%
Male gamatophyte	60	3	**M60-3-3**	3,878,302	297,321,323	76.66	3,699,151	69,650	10.86	10,340,382	0.3375%
Seed	30	-	**S30-1**	8,864,974	676,821,130	76.35	8,482,995	91,537	11.56	13,304,446	0.4342%
Seed	30	-	**S30-2**	6,160,328	471,450,093	76.53	5,919,412	76,384	11.85	10,917,848	0.3563%
Seed	30	-	**S30-3**	9,972,088	761,712,077	76.38	9,613,688	93,701	12.14	13,403,507	0.4375%
Seed	30	-	**S30-4**	2,558,410	196,260,826	76.71	2,462,191	51,129	11.77	7,185,109	0.2345%
Seed	60	-	**S60-1**	11,001,120	843,436,197	76.67	10,573,805	96,907	12.40	13,710,256	0.4475%

Irradiated tissue	Dose (Gy)	Flower stage	Individual ID	Sum of trimmed reads	Total length of trimmed reads (bp)	Average length of trimmed reads (bp)	No. mapped reads	No. mapped region	Median depth of mapped region	Total length of mapped region (bp)	Reference genome coverage (%)
Seed	60	-	**S60-2**	7,290,230	558,252,231	76.58	7,014,154	81,362	12.64	11,558,116	0.3772%
Seed	60	-	**S60-3**	4,649,916	356,111,875	76.58	4,477,577	72,613	11.38	10,327,861	0.3371%
Seed	60	-	**S60-4**	11,842,522	907,849,413	76.66	11,379,290	108,735	12.52	15,154,838	0.4946%
Seed	120	-	**S120-1**	13,011,772	979,995,497	75.32	12,510,986	112,461	12.36	15,869,022	0.5179%
Seed	120	-	**S120-2**	5,948,108	452,798,015	76.12	5,694,505	78,359	11.76	11,351,790	0.3705%
Seed	120	-	**S120-3**	7,661,368	583,817,166	76.20	7,292,669	83,488	11.88	12,034,907	0.3928%
Seed	120	-	**S120-4**	10,098,802	773,731,218	76.62	9,722,878	94,311	12.05	13,332,723	0.4352%

Table S4. Statistics for the genotype-by-sequencing (GBS) analysis performed for analysis of recombination frequency

Irradiated tissue	Dose (Gy)	Flower stage	Individual ID	Sum of trimmed reads	Total length of trimmed reads (bp)	Average length of trimmed reads (bp)	No. mapped reads	No. mapped region	Median depth of mapped region	Total length of mapped region (bp)	Reference Genome coverage (%)
-	Not-irradiated	-	**BC1-1**	4,192,216	351,927,569	83.95	3,251,214	90,079	7.31	14,949,273	0.4879%
-	Not-irradiated	-	**BC1-2**	3,356,024	280,753,214	83.66	2,141,601	72,932	6.67	11,989,315	0.3913%
-	Not-irradiated	-	**BC1-3**	6,227,440	521,774,518	83.79	2,473,261	75,434	7.30	12,279,186	0.4008%
-	Not-irradiated	-	**BC1-4**	4,173,808	352,804,024	84.53	2,911,967	93,214	6.93	15,676,189	0.5116%
-	Not-irradiated	-	**BC1-5**	3,563,862	299,796,371	84.12	3,097,107	98,605	6.80	16,978,009	0.5541%
Female gamatophyte	15	0	**15-0-1**	6,524,318	543,192,100	83.26	5,265,550	113,605	8.13	18,960,633	0.6188%
Female gamatophyte	15	0	**15-0-2**	4,656,802	391,842,420	84.14	2,915,309	83,136	7.59	13,596,978	0.4438%
Female gamatophyte	15	0	**15-0-3**	10,465,282	878,752,709	83.97	3,470,615	92,604	7.46	15,343,109	0.5008%
Female gamatophyte	15	0	**15-0-4**	5,383,416	453,353,327	84.21	3,835,279	106,513	7.33	18,530,528	0.6048%
Female gamatophyte	15	0	**15-0-5**	3,832,666	322,797,450	84.22	3,246,478	98,772	7.08	16,913,639	0.5520%
Female gamatophyte	15	0	**15-0-6**	3,709,924	314,040,768	84.65	2,251,343	76,308	6.81	12,613,232	0.4117%
Female gamatophyte	15	0	**15-0-7**	2,299,056	194,161,138	84.45	1,528,498	61,569	6.34	9,811,778	0.3202%
Female gamatophyte	15	0	**15-0-8**	4,760,616	401,205,310	84.28	3,525,855	104,164	7.05	17,894,986	0.5841%
Female gamatophyte	15	0	**15-0-9**	5,936,562	497,688,950	83.83	4,463,488	105,767	7.81	17,675,865	0.5769%
Female gamatophyte	15	0	**15-0-10**	2,495,206	209,994,486	84.16	1,649,651	61,869	6.68	9,920,670	0.3238%

Irradiated tissue	Dose (Gy)	Flower stage	Individual ID	Sum of trimmed reads	Total length of trimmed reads (bp)	Average length of trimmed reads (bp)	No. mapped reads	No. mapped region	Median depth of mapped region	Total length of mapped region (bp)	Reference Genome coverage (%)
Female gamatophyte	15	1	**15-1-1**	4,918,004	412,367,669	83.85	4,456,211	117,638	7.28	19,785,043	0.6458%
Female gamatophyte	15	1	**15-1-2**	1,967,222	165,367,883	84.06	1,460,000	61,038	6.11	9,753,068	0.3183%
Female gamatophyte	15	1	**15-1-3**	3,498,532	293,348,459	83.85	1,387,265	57,084	6.07	9,046,277	0.2953%
Female gamatophyte	15	1	**15-1-4**	3,743,714	317,422,248	84.79	3,374,089	101,887	7.20	17,397,001	0.5678%
Female gamatophyte	15	1	**15-1-5**	4,946,540	416,229,649	84.15	2,504,719	89,754	6.38	15,099,813	0.4928%
Female gamatophyte	15	1	**15-1-6**	3,709,552	310,301,651	83.65	2,997,156	101,895	6.58	17,650,807	0.5761%
Female gamatophyte	15	1	**15-1-7**	4,892,568	408,493,007	83.49	4,540,751	113,849	7.41	19,367,440	0.6321%
Female gamatophyte	15	1	**15-1-8**	2,773,702	231,847,973	83.59	2,554,159	87,854	6.72	14,625,636	0.4774%
Female gamatophyte	15	1	**15-1-9**	4,450,700	372,916,712	83.79	2,755,992	81,037	7.38	13,270,107	0.4331%
Female gamatophyte	15	1	**15-1-10**	3,670,252	306,277,437	83.45	2,390,042	84,010	6.56	14,072,591	0.4593%
Female gamatophyte	15	1	**15-1-11**	5,326,712	443,522,861	83.26	2,170,633	77,395	6.00	12,853,075	0.4195%
Female gamatophyte	15	2	**15-2-1**	2,002,564	167,113,080	83.45	1,709,532	67,021	6.06	10,696,359	0.3491%
Female gamatophyte	15	2	**15-2-2**	6,990,820	583,561,324	83.48	2,965,173	92,473	6.79	15,786,875	0.5153%
Female gamatophyte	15	2	**15-2-3**	3,148,842	263,977,281	83.83	2,694,710	87,078	6.95	14,485,011	0.4728%
Female gamatophyte	15	2	**15-2-4**	3,752,688	313,588,981	83.56	3,383,861	94,342	6.91	15,399,178	0.5026%
Female gamatophyte	30	0	**30-0-1**	3,304,974	275,816,942	83.46	1,811,758	65,942	6.82	10,408,043	0.3397%

Irradiated tissue	Dose (Gy)	Flower stage	Individual ID	Sum of trimmed reads	Total length of trimmed reads (bp)	Average length of trimmed reads (bp)	No. mapped reads	No. mapped region	Median depth of mapped region	Total length of mapped region (bp)	Reference Genome coverage (%)
Female gamatophyte	30	0	**30-0-2**	8,664,084	722,471,031	83.39	4,228,228	103,968	7.83	17,217,749	0.5620%
Female gamatophyte	30	0	**30-0-3**	6,973,286	583,182,797	83.63	3,395,964	89,339	7.90	14,679,895	0.4791%
Female gamatophyte	30	0	**30-0-4**	9,144,336	761,957,216	83.33	2,044,410	70,226	6.95	10,936,528	0.3570%
Female gamatophyte	30	0	**30-0-5**	6,865,898	572,987,250	83.45	1,750,862	62,204	6.98	9,798,050	0.3198%
Female gamatophyte	30	0	**30-0-6**	10,640,430	880,341,869	82.74	2,668,907	85,001	7.03	14,004,998	0.4571%
Female gamatophyte	30	1	**30-1-1**	9,392,132	780,228,273	83.07	3,037,911	89,390	7.22	14,899,222	0.4863%
Female gamatophyte	30	1	**30-1-2**	5,594,648	465,571,491	83.22	4,445,408	110,338	7.65	18,565,505	0.6059%
Female gamatophyte	30	1	**30-1-3**	9,450,052	787,332,449	83.32	5,216,409	105,589	8.89	16,940,324	0.5529%
Female gamatophyte	30	1	**30-1-4**	8,394,588	698,800,163	83.24	7,367,218	127,755	8.80	20,783,273	0.6783%

Table S5. Information of single base substitutions (SBSs) detected by the genotype-by-sequencing (GBS) analysis performed for analysis of mutation frequency

Individual ID	Chromosome ID	Position on chromosome	Genotype in original line	Genotype in mutant line	Feature
**F15-3-1**	chr01	31,179,315	C/C	T/C	Intergenic
**F15-3-1**	chr01	252,597,481	G/G	G/T	Intron
**F15-3-1**	chr02	42,126,486	T/T	T/C	Exon
**F15-3-1**	chr02	167,245,892	C/C	T/C	Exon
**F15-3-1**	chr03	19,976,142	G/G	A/G	Intergenic
**F15-3-1**	chr04	14,768,196	A/A	A/G	Exon
**F15-3-1**	chr04	15,843,937	G/G	A/G	Exon
**F15-3-1**	chr04	187,935,260	A/A	C/C	Intergenic
**F15-3-1**	chr04	211,805,160	C/C	T/C	Intergenic
**F15-3-1**	chr04	228,851,522	A/A	A/T	Exon
**F15-3-1**	chr04	231,091,884	G/G	A/G	Exon
**F15-3-1**	chr05	104,624,257	C/C	A/A	Intergenic
**F15-3-1**	chr05	238,080,642	C/C	T/C	Exon
**F15-3-1**	chr07	192,867,236	T/T	T/C	Exon
**F15-3-1**	chr08	119,888,730	A/A	A/G	Intergenic
**F15-3-1**	chr08	135,652,647	C/C	T/C	Exon
**F15-3-1**	chr09	839,523	C/C	T/T	Intron
**F15-3-1**	chr09	14,612,738	G/G	A/G	Exon
**F15-3-1**	chr09	255,757,721	C/C	T/C	Exon
**F15-3-1**	chr09	270,175,920	C/C	T/C	Intergenic
**F15-3-1**	chr11	2,217,415	A/A	A/G	Intron
**F15-3-1**	chr11	2,339,033	A/A	A/G	Exon
**F15-3-1**	chr11	235,721,562	A/A	A/G	Intergenic
**F15-3-1**	chr12	8,395,940	T/T	T/C	Exon
**F15-3-1**	chr12	246,065,360	G/G	G/T	Intron
**F15-3-1**	PGAv.1.6.scaffold1190	690,138	A/A	A/T	Intergenic
**F15-3-1**	PGAv.1.6.scaffold1222	631,813	C/C	T/C	Exon
**F15-3-1**	PGAv.1.6.scaffold1389	368,629	A/A	A/G	Intron
**F15-3-1**	PGAv.1.6.scaffold2128	7,921	C/C	G/C	
**F15-3-2**	chr01	199,473,940	G/G	A/G	Exon
**F15-3-2**	chr02	137,949,857	A/A	A/G	Intergenic
**F15-3-2**	chr02	142,803,573	C/C	T/C	Exon
**F15-3-2**	chr02	150,484,423	C/C	T/C	Exon
**F15-3-2**	chr02	155,779,906	A/A	A/G	Intron
**F15-3-2**	chr02	165,842,595	T/T	A/T	Exon
**F15-3-2**	chr02	167,544,153	T/T	T/C	Intron
**F15-3-2**	chr03	21,781,377	G/G	G/C	Exon
**F15-3-2**	chr03	146,544,978	A/A	A/G	Intergenic
**F15-3-2**	chr04	237,809,285	G/G	G/T	Intergenic
**F15-3-2**	chr04	239,312,658	C/C	T/C	Exon
**F15-3-2**	chr05	2,355,579	T/T	T/C	Exon
**F15-3-2**	chr08	126,462,893	G/G	G/T	Exon
**F15-3-2**	chr09	266,004,126	A/A	A/T	Intron
**F15-3-2**	chr10	194,597,995	G/G	A/G	Exon
**F15-3-2**	chr10	231,647,350	C/C	T/C	Intergenic
**F15-3-2**	chr11	69,336,922	A/A	A/G	Intergenic
**F15-3-2**	PGAv.1.6.scaffold523	1,262,438	G/G	A/G	Exon
**F15-3-2**	PGAv.1.6.scaffold855	828,213	T/T	T/C	Exon
**F15-3-2**	PGAv.1.6.scaffold1235	634,418	A/A	A/T	Exon
**F15-3-2**	PGAv.1.6.scaffold1447	147,196	G/G	A/G	Intergenic
**F15-3-2**	PGAv.1.6.scaffold4474	801	A/A	A/T	
**F15-3-2**	PGAv.1.6.scaffold30381	217	A/A	A/T	
**F15-3-3**	chr01	12,615,554	G/G	A/G	Exon
**F15-3-3**	chr01	69,193,152	A/A	A/T	Intron
**F15-3-3**	chr03	11,884,630	A/A	A/G	Intron
**F15-3-3**	chr06	226,820,262	C/C	T/C	Intergenic
**F15-3-3**	chr07	2,422,042	G/G	A/G	Intergenic
**F15-3-3**	chr07	87,271,976	A/A	A/G	Intron

Individual ID	Chromosome ID	Position on chromosome	Genotype in original line	Genotype in mutant line	Feature
**F15-3-3**	chr09	5,431,083	C/C	T/C	Intron
**F15-3-3**	chr10	158,403,390	T/T	A/T	Exon
**F15-3-3**	chr10	231,645,661	T/T	A/T	Intergenic
**F15-3-3**	chr12	242,711,230	C/C	G/C	Exon
**F30-2-1**	chr01	92,698,582	T/T	A/T	Exon
**F30-2-1**	chr01	233,975,002	G/G	A/G	Intergenic
**F30-2-1**	chr03	27,704,950	A/A	A/G	Intergenic
**F30-2-1**	chr05	164,986,140	G/G	A/G	Intron
**F30-2-1**	chr05	238,022,249	A/A	A/G	Exon
**F30-2-1**	chr05	238,022,268	C/C	T/C	Exon
**F30-2-1**	chr06	121,971,282	G/G	A/G	Exon
**F30-2-1**	chr07	245,837,695	G/G	A/G	Intron
**F30-2-1**	chr09	2,309,324	G/G	G/T	Exon
**F30-2-1**	chr09	201,989,255	A/A	A/G	Exon
**F30-2-1**	chr11	64,720,815	T/T	G/T	Intergenic
**F30-2-1**	chr11	135,880,480	G/G	A/G	Intergenic
**F30-2-1**	chr11	260,430,805	G/G	A/G	Intergenic
**F30-2-1**	chr12	172,124,733	G/G	A/G	Exon
**F30-2-1**	chr12	179,725,143	G/G	A/A	Intergenic
**F30-2-1**	chr12	228,127,345	G/G	A/G	Intergenic
**F30-2-1**	chr12	250,473,211	A/A	A/G	Intron
**F30-2-1**	PGAv.1.6.scaffold459	98,268	A/A	A/T	Intergenic
**F30-2-1**	PGAv.1.6.scaffold959	746,940	C/C	T/C	Exon
**F30-2-1**	PGAv.1.6.scaffold1190	37,792	G/G	A/G	Intron
**F30-2-1**	PGAv.1.6.scaffold1231	296,546	T/T	A/T	Exon
**F30-2-1**	PGAv.1.6.scaffold1412	176,249	C/C	T/C	Intron
**F30-2-1**	PGAv.1.6.scaffold1715	225,924	C/C	A/C	Intron
**F30-2-2**	chr01	9,009,364	C/C	T/C	Intron
**F30-2-2**	chr01	306,090,279	A/A	A/G	Intergenic
**F30-2-2**	chr03	201,189,308	A/A	A/G	Intergenic
**F30-2-2**	chr03	267,327,599	T/T	A/T	Intron
**F30-2-2**	chr03	267,414,666	A/A	A/T	Exon
**F30-2-2**	chr03	273,945,289	T/T	G/T	Exon
**F30-2-2**	chr03	277,198,646	A/A	A/G	Exon
**F30-2-2**	chr08	137,903,796	A/A	A/G	Intergenic
**F30-2-2**	chr12	235,584,697	G/G	A/G	Intron
**F30-2-2**	PGAv.1.6.scaffold1155	297,952	T/T	T/C	Intron
**F30-2-2**	PGAv.1.6.scaffold30381	217	A/A	A/T	
**F30-2-3**	chr01	39,546,644	C/C	A/C	Intergenic
**F30-2-3**	chr02	146,860,716	A/A	A/G	Intergenic
**F30-2-3**	chr02	164,705,326	T/T	A/T	Intergenic
**F30-2-3**	chr03	239,119,752	T/T	T/C	Intron
**F30-2-3**	chr04	7,960,631	T/T	A/T	Intergenic
**F30-2-3**	chr04	14,780,485	T/T	T/C	Exon
**F30-2-3**	chr04	140,462,973	C/C	T/C	Intergenic
**F30-2-3**	chr06	74,397,448	C/C	A/C	Intergenic
**F30-2-3**	chr11	69,336,922	A/A	A/G	Intergenic
**F30-2-3**	PGAv.1.6.scaffold606	1,488,884	C/C	T/C	Intron
**F30-2-3**	PGAv.1.6.scaffold1041	85,807	A/A	A/T	Exon
**F30-3-1**	chr01	44,177,798	G/G	A/G	Intergenic
**F30-3-1**	chr01	131,198,013	G/G	A/G	Intergenic
**F30-3-1**	chr01	256,287,860	G/G	A/G	Exon
**F30-3-1**	chr01	259,340,711	G/G	G/T	Intergenic
**F30-3-1**	chr02	114,683,867	A/A	A/T	Exon
**F30-3-1**	chr02	146,085,931	G/G	G/T	Intron
**F30-3-1**	chr02	150,579,518	C/C	T/C	Intron
**F30-3-1**	chr02	168,385,713	T/T	G/T	Intron
**F30-3-1**	chr03	10,421,033	A/A	A/G	Intergenic
**F30-3-1**	chr03	20,829,604	G/G	G/T	Intron
**F30-3-1**	chr04	9,827,551	G/G	A/G	Exon
**F30-3-1**	chr04	101,748,488	C/C	T/C	Intergenic

Individual ID	Chromosome ID	Position on chromosome	Genotype in original line	Genotype in mutant line	Feature
**F30-3-1**	chr06	230,080,836	C/C	A/C	Exon
**F30-3-1**	chr07	225,994,952	G/G	A/G	Intergenic
**F30-3-1**	chr07	234,413,802	G/G	A/G	Intergenic
**F30-3-1**	chr10	189,078,432	T/T	A/T	Exon
**F30-3-1**	chr12	246,706,526	C/C	T/C	Intron
**F30-3-1**	PGAv.1.6.scaffold866	182,271	C/C	T/C	Exon
**F30-3-1**	PGAv.1.6.scaffold1721	187,438	T/T	T/C	Exon
**F30-3-1**	PGAv.1.6.scaffold1909	87,730	G/G	G/T	Intergenic
**F30-3-1**	PGAv.1.6.scaffold2076	17,280	C/C	T/C	Intergenic
**F30-3-2**	chr01	252,597,481	G/G	G/T	Intron
**F30-3-2**	chr05	2,355,579	T/T	T/C	Exon
**F30-3-2**	chr05	37,576,723	C/C	A/C	Intron
**F30-3-2**	chr06	228,091,237	G/G	A/G	Exon
**F30-3-2**	chr06	237,537,697	A/A	A/G	Intergenic
**F30-3-2**	chr08	83,420,421	G/G	A/G	Intergenic
**F30-3-2**	chr10	228,552,975	A/A	A/G	Intron
**F30-3-2**	chr10	232,829,799	C/C	T/C	Intergenic
**F30-3-2**	chr11	983,271	T/T	T/C	Intergenic
**F30-3-2**	chr12	2,463,128	C/C	T/C	Intergenic
**F30-3-2**	PGAv.1.6.scaffold798	56,254	G/G	G/T	Intron
**F30-3-2**	PGAv.1.6.scaffold2211	33,630	C/C	T/C	Intron
**F30-3-3**	chr01	195,645,456	T/T	G/T	Exon
**F30-3-3**	chr02	5,982,491	G/G	A/G	Exon
**F30-3-3**	chr02	131,717,791	T/T	T/C	Intergenic
**F30-3-3**	chr03	1,866,136	C/C	T/C	Exon
**F30-3-3**	chr03	175,970,565	G/G	G/T	Intron
**F30-3-3**	chr03	253,248,225	G/G	A/G	Exon
**F30-3-3**	chr03	273,987,862	C/C	A/C	Intergenic
**F30-3-3**	chr03	282,758,750	T/T	A/T	Intron
**F30-3-3**	chr04	218,640,840	C/C	T/C	Intergenic
**F30-3-3**	chr05	111,067,249	G/G	A/G	Intergenic
**F30-3-3**	chr05	214,448,156	A/A	A/G	Intergenic
**F30-3-3**	chr05	238,049,327	A/A	A/T	Intergenic
**F30-3-3**	chr05	238,212,913	A/A	A/C	Intergenic
**F30-3-3**	chr06	228,722,898	T/T	T/C	Exon
**F30-3-3**	chr08	68,958,243	T/T	T/C	Intergenic
**F30-3-3**	chr10	201,541,798	C/C	T/C	Exon
**F30-3-3**	chr11	90,620,891	T/T	T/C	Intergenic
**F30-3-3**	chr11	151,470,634	C/C	T/C	Intergenic
**F30-3-3**	chr11	252,650,559	C/C	T/C	Intron
**F30-3-3**	PGAv.1.6.scaffold504	666,800	T/T	T/C	Intergenic
**F30-3-3**	PGAv.1.6.scaffold1715	225,985	T/T	A/T	Intron
**F60-1-1**	chr01	198,080,026	G/G	A/G	Intergenic
**F60-1-1**	chr01	297,565,393	A/A	A/G	Exon
**F60-1-1**	chr06	112,352,865	A/A	A/T	Intergenic
**F60-1-1**	chr06	213,548,889	T/T	G/T	Intergenic
**F60-1-1**	chr07	94,815,403	T/T	T/C	Intron
**F60-1-1**	chr08	1,161,218	G/G	G/T	Exon
**F60-1-1**	chr08	141,348,333	G/G	A/G	Intron
**F60-1-1**	chr09	839,523	C/C	T/C	Intron
**F60-1-1**	chr09	158,159,986	C/C	T/C	Intergenic
**F60-1-1**	chr11	69,336,922	A/A	A/G	Intergenic
**F60-1-2**	chr01	24,408,510	C/C	T/C	Intron
**F60-1-2**	chr01	52,222,325	A/A	A/G	Intergenic
**F60-1-2**	chr01	246,694,956	A/A	A/T	Exon
**F60-1-2**	chr01	292,573,844	G/G	A/G	Exon
**F60-1-2**	chr01	303,744,145	A/A	A/G	Exon
**F60-1-2**	chr02	142,169,985	C/C	A/A	Exon
**F60-1-2**	chr02	159,270,195	C/C	T/C	Exon
**F60-1-2**	chr03	101,364,958	C/C	T/C	Intron
**F60-1-2**	chr03	176,649,274	G/G	A/G	Intron

Individual ID	Chromosome ID	Position on chromosome	Genotype in original line	Genotype in mutant line	Feature
**F60-1-2**	chr03	270,320,843	A/A	A/T	Exon
**F60-1-2**	chr03	278,189,500	G/G	A/G	Intron
**F60-1-2**	chr05	95,145,540	C/C	A/C	Intergenic
**F60-1-2**	chr05	166,733,757	T/T	T/C	Exon
**F60-1-2**	chr05	226,913,949	A/A	A/G	Intron
**F60-1-2**	chr06	3,468,329	C/C	A/C	Exon
**F60-1-2**	chr07	4,689,703	T/T	T/C	Exon
**F60-1-2**	chr07	108,007,179	T/T	A/T	Intergenic
**F60-1-2**	chr08	36,050,701	G/G	A/G	Intergenic
**F60-1-2**	chr09	143,614,720	A/A	A/G	Intron
**F60-1-2**	chr09	255,180,810	G/G	G/T	Exon
**F60-1-2**	chr09	264,347,919	T/T	C/C	Intergenic
**F60-1-2**	chr10	124,269,707	C/C	G/C	Intergenic
**F60-1-2**	chr10	233,059,280	T/T	T/C	Intergenic
**F60-1-2**	chr11	1,667,095	A/A	A/T	Exon
**F60-1-2**	chr11	2,669,898	A/A	A/T	Exon
**F60-1-2**	chr12	233,860,615	A/A	A/T	Intron
**F60-1-2**	PGAv.1.6.scaffold929	384,280	G/G	A/G	Intergenic
**F60-1-2**	PGAv.1.6.scaffold1335	203,223	T/T	T/C	Exon
**F60-1-2**	PGAv.1.6.scaffold1379	187,499	C/C	A/C	Intergenic
**F60-1-2**	PGAv.1.6.scaffold1652	140,166	T/T	G/T	Intergenic
**F60-1-2**	PGAv.1.6.scaffold1768	97,771	C/C	A/C	Intergenic
**F60-1-2**	PGAv.1.6.scaffold2708	9,073	G/G	A/G	Intergenic
**F60-1-2**	PGAv.1.6.scaffold2708	9,074	C/C	T/C	Intergenic
**F60-1-2**	PGAv.1.6.scaffold3402	2,398	C/C	A/C	
**F60-1-3**	chr01	2,389,530	A/A	A/T	Intron
**F60-1-3**	chr01	58,016,244	G/G	A/G	Exon
**F60-1-3**	chr01	228,798,068	T/T	A/T	Exon
**F60-1-3**	chr01	288,754,928	C/C	T/C	Intergenic
**F60-1-3**	chr02	119,270,327	A/A	A/T	Intergenic
**F60-1-3**	chr02	138,448,557	G/G	A/G	Intergenic
**F60-1-3**	chr03	19,153,505	T/T	G/T	Intergenic
**F60-1-3**	chr03	23,870,573	C/C	T/C	Intergenic
**F60-1-3**	chr03	232,704,193	G/G	A/G	Intron
**F60-1-3**	chr04	16,241,714	G/G	A/G	Intergenic
**F60-1-3**	chr05	2,355,579	T/T	T/C	Exon
**F60-1-3**	chr05	236,808,273	T/T	T/C	Intron
**F60-1-3**	chr06	68,885,220	A/A	A/G	Intergenic
**F60-1-3**	chr06	201,138,416	T/T	T/C	Intergenic
**F60-1-3**	chr06	220,263,930	C/C	T/C	Intron
**F60-1-3**	chr06	237,979,108	G/G	A/G	Intergenic
**F60-1-3**	chr07	89,656,247	T/T	T/C	Intergenic
**F60-1-3**	chr08	97,952,620	T/T	A/T	Intron
**F60-1-3**	chr09	229,382,793	C/C	T/C	Intergenic
**F60-1-3**	chr10	138,278,555	G/G	A/G	Intergenic
**F60-1-3**	chr10	233,265,340	T/T	T/C	Exon
**F60-1-3**	chr11	2,339,033	A/A	A/G	Exon
**F60-1-3**	chr11	252,548,460	G/G	A/G	Intron
**F60-1-3**	chr11	265,989,633	A/A	A/T	Intergenic
**F60-1-3**	chr12	146,291,180	G/G	A/G	Intron
**F60-1-3**	PGAv.1.6.scaffold1273	335,367	C/C	T/C	Intergenic
**F60-1-3**	PGAv.1.6.scaffold1751	108,156	T/T	T/C	Intron
**F60-1-3**	PGAv.1.6.scaffold2076	17,280	C/C	T/C	Intergenic
**F60-1-3**	PGAv.1.6.scaffold2076	17,330	A/A	A/G	Intergenic
**F60-2-1**	chr01	4,252,082	G/G	A/G	Intergenic
**F60-2-1**	chr01	59,485,658	C/C	A/C	Intergenic
**F60-2-1**	chr01	256,927,123	T/T	T/C	Intergenic
**F60-2-1**	chr02	160,170,460	C/C	T/T	Exon
**F60-2-1**	chr03	7,732,128	T/T	A/T	Exon
**F60-2-1**	chr03	85,700,684	A/A	A/T	Intron
**F60-2-1**	chr04	218,311,937	A/A	A/G	Exon

Individual ID	Chromosome ID	Position on chromosome	Genotype in original line	Genotype in mutant line	Feature
**F60-2-1**	chr04	234,395,585	G/G	A/A	Exon
**F60-2-1**	chr04	234,395,627	A/A	G/G	Exon
**F60-2-1**	chr05	1,662,097	A/A	A/T	Intergenic
**F60-2-1**	chr05	9,303,819	C/C	T/C	Intergenic
**F60-2-1**	chr06	8,482,533	C/C	A/C	Exon
**F60-2-1**	chr06	142,363,663	C/C	T/C	Intergenic
**F60-2-1**	chr06	231,552,136	C/C	T/C	Exon
**F60-2-1**	chr07	127,101,577	A/A	A/G	Exon
**F60-2-1**	chr07	248,727,955	A/A	A/G	Intron
**F60-2-1**	chr09	5,824,516	C/C	T/C	Intergenic
**F60-2-1**	chr09	27,405,784	C/C	T/C	Intergenic
**F60-2-1**	chr09	220,470,414	G/G	A/G	Intergenic
**F60-2-1**	chr10	150,903,225	A/A	A/C	Intergenic
**F60-2-1**	chr11	147,171,722	A/A	A/G	Exon
**F60-2-1**	chr12	232,459	T/T	T/C	Exon
**F60-2-1**	chr12	142,177,465	T/T	T/C	Exon
**F60-2-1**	PGAv.1.6.scaffold942	194,327	C/C	A/C	Intergenic
**F60-2-2**	chr01	3,284,488	T/T	T/C	Exon
**F60-2-2**	chr01	20,134,209	C/C	T/C	Intergenic
**F60-2-2**	chr01	192,588,736	G/G	A/G	Intron
**F60-2-2**	chr02	50,429,993	G/G	A/G	Intergenic
**F60-2-2**	chr02	152,289,060	C/C	T/C	Intergenic
**F60-2-2**	chr03	10,362,442	G/G	G/T	Intergenic
**F60-2-2**	chr03	10,362,515	T/T	T/C	Intergenic
**F60-2-2**	chr03	10,362,547	A/A	A/G	Intergenic
**F60-2-2**	chr03	94,926,781	C/C	A/C	Intergenic
**F60-2-2**	chr04	187,935,260	A/A	A/C	Intergenic
**F60-2-2**	chr05	123,535,557	T/T	A/A	Intergenic
**F60-2-2**	chr06	207,787,807	C/C	T/C	Intron
**F60-2-2**	chr06	229,091,401	G/G	G/C	Intron
**F60-2-2**	chr06	235,200,186	C/C	T/C	Exon
**F60-2-2**	chr08	74,316,750	T/T	T/C	Intergenic
**F60-2-2**	chr09	68,475,148	C/C	T/C	Intergenic
**F60-2-2**	chr09	266,760,850	C/C	G/C	Exon
**F60-2-2**	chr09	268,771,675	C/C	A/C	Exon
**F60-2-2**	chr10	17,574,466	T/T	T/C	Intron
**F60-2-2**	chr10	125,720,327	A/A	A/G	Intergenic
**F60-2-2**	chr11	219,486,293	A/A	A/T	Exon
**F60-2-2**	chr12	4,270,427	G/G	A/G	Intron
**F60-2-2**	chr12	156,292,873	T/T	A/T	Exon
**F60-2-2**	chr12	205,836,423	A/A	A/T	Exon
**F60-2-3**	chr01	63,034,308	C/C	T/C	Intergenic
**F60-2-3**	chr01	252,597,481	G/G	G/T	Intron
**F60-2-3**	chr02	168,808,528	A/A	A/T	Exon
**F60-2-3**	chr03	6,172,126	T/T	A/T	Exon
**F60-2-3**	chr04	1,025,096	C/C	A/C	Exon
**F60-2-3**	chr04	203,182,158	A/A	A/T	Exon
**F60-2-3**	chr05	2,355,579	T/T	T/C	Exon
**F60-2-3**	chr05	104,869,042	T/T	T/C	Intergenic
**F60-2-3**	chr07	2,422,042	G/G	A/G	Intergenic
**F60-2-3**	chr07	20,184,641	A/A	A/T	Intergenic
**F60-2-3**	chr07	245,984,275	T/T	G/T	Intergenic
**F60-2-3**	chr09	21,532,381	C/C	T/C	Intergenic
**F60-2-3**	chr09	195,271,694	C/C	A/C	Intergenic
**F60-2-3**	chr11	51,594,630	C/C	T/C	Intergenic
**F60-2-3**	chr11	57,103,399	C/C	A/C	Exon
**F60-2-3**	chr12	81,348,095	C/C	T/C	Intergenic
**F60-2-3**	chr12	244,875,334	T/T	A/T	Intergenic
**F60-2-3**	PGAv.1.6.scaffold1110	339,863	G/G	A/G	Intron
**F60-2-3**	PGAv.1.6.scaffold1544	298,975	C/C	G/C	Intergenic
**F60-2-3**	PGAv.1.6.scaffold1889	90,683	G/G	A/G	Exon

Individual ID	Chromosome ID	Position on chromosome	Genotype in original line	Genotype in mutant line	Feature
**F60-2-3**	PGAv.1.6.scaffold1889	90,691	A/A	A/C	Exon
**F60-3-1**	chr02	163,448,775	C/C	A/C	Intergenic
**F60-3-1**	chr03	20,829,621	G/G	G/T	Intron
**F60-3-1**	chr04	17,587,272	C/C	T/C	Intergenic
**F60-3-1**	chr05	2,355,579	T/T	T/C	Exon
**F60-3-1**	chr10	119,822,547	G/G	A/G	Intergenic
**F60-3-1**	PGAv.1.6.scaffold1339	205,533	C/C	A/C	Intergenic
**F60-3-1**	PGAv.1.6.scaffold1339	205,613	C/C	T/C	Intergenic
**F60-3-2**	chr03	10,421,035	A/A	A/T	Intergenic
**F60-3-2**	chr03	137,535,478	A/A	A/G	Exon
**F60-3-2**	chr05	9,166,919	T/T	T/C	Intron
**F60-3-2**	chr05	230,479,300	A/A	A/G	Intron
**F60-3-2**	chr05	233,005,700	G/G	G/T	Exon
**F60-3-2**	chr05	233,596,910	G/G	A/G	Intergenic
**F60-3-2**	chr08	25,599,514	G/G	A/G	Intergenic
**F60-3-2**	chr08	139,450,000	A/A	A/G	Intron
**F60-3-2**	chr09	37,922,658	G/G	G/T	Intergenic
**F60-3-2**	chr10	217,396,446	T/T	T/C	Exon
**F60-3-2**	chr11	101,520,464	C/C	T/C	Exon
**F60-3-2**	chr12	84,974,922	C/C	T/C	Intergenic
**F60-3-2**	PGAv.1.6.scaffold1703	117,809	C/C	T/C	Exon
**F60-3-2**	PGAv.1.6.scaffold2162	4,321	G/G	A/G	
**F60-3-3**	chr01	193,243,020	T/T	T/C	Intergenic
**F60-3-3**	chr01	193,243,060	C/C	T/C	Intergenic
**F60-3-3**	chr02	161,043,198	C/C	T/C	Exon
**F60-3-3**	chr03	9,469,543	A/A	A/T	Intron
**F60-3-3**	chr03	29,261,517	G/G	A/G	Exon
**F60-3-3**	chr03	257,031,114	T/T	T/C	Intergenic
**F60-3-3**	chr04	237,457,485	A/A	A/T	Intron
**F60-3-3**	chr05	2,355,579	T/T	T/C	Exon
**F60-3-3**	chr05	225,398,209	C/C	T/C	Exon
**F60-3-3**	chr06	10,928,662	G/G	A/G	Intergenic
**F60-3-3**	chr06	230,823,732	G/G	A/G	Exon
**F60-3-3**	chr07	242,123,061	T/T	A/T	Intergenic
**F60-3-3**	chr07	245,593,474	T/T	T/C	Intergenic
**F60-3-3**	chr07	245,593,534	C/C	G/C	Intergenic
**F60-3-3**	chr07	245,593,558	A/A	A/G	Intergenic
**F60-3-3**	chr08	5,057,503	T/T	A/T	Exon
**F60-3-3**	chr08	96,779,682	G/G	A/G	Intron
**F60-3-3**	chr09	135,422,738	T/T	T/C	Intergenic
**F60-3-3**	chr12	51,142,554	T/T	T/C	Intron
**F60-3-3**	PGAv.1.6.scaffold906	822,414	C/C	A/C	Exon
**F60-3-3**	PGAv.1.6.scaffold1006	872,914	G/G	A/G	Intron
**M15-3-1**	chr02	115,997,158	T/T	T/C	Intron
**M15-3-1**	chr02	148,565,440	G/G	G/T	Exon
**M15-3-1**	chr03	26,844,949	A/A	A/G	Exon
**M15-3-1**	chr04	101,748,488	C/C	T/C	Intergenic
**M15-3-1**	chr05	104,869,110	C/C	G/C	Intergenic
**M15-3-1**	chr05	228,649,666	C/C	G/C	Intergenic
**M15-3-1**	chr07	43,825,090	T/T	T/C	Intron
**M15-3-1**	chr09	270,906,067	G/G	A/G	Intergenic
**M15-3-1**	chr11	9,564,194	A/A	A/G	Exon
**M15-3-1**	chr11	16,461,161	A/A	A/T	Intergenic
**M15-3-1**	PGAv.1.6.scaffold1248	538,161	T/T	A/T	Intergenic
**M15-3-2**	chr01	203,629,444	A/A	A/T	Intergenic
**M15-3-2**	chr03	234,175,977	C/C	T/C	Intron
**M15-3-2**	chr03	273,820,091	A/A	A/T	Intron
**M15-3-2**	chr04	961,654	T/T	A/T	Exon
**M15-3-2**	chr04	40,743,351	G/G	A/G	Intergenic
**M15-3-2**	chr04	202,352,385	G/G	A/G	Intergenic
**M15-3-2**	chr04	209,235,635	G/G	G/T	Intron

Individual ID	Chromosome ID	Position on chromosome	Genotype in original line	Genotype in mutant line	Feature
**M15-3-2**	chr05	222,547,607	T/T	T/C	Exon
**M15-3-2**	chr05	222,547,616	A/A	A/G	Exon
**M15-3-2**	chr07	54,130,710	C/C	T/C	Intergenic
**M15-3-2**	chr08	135,224,568	A/A	A/G	Intergenic
**M15-3-2**	chr09	4,960,978	G/G	A/G	Intergenic
**M15-3-2**	chr09	166,406,237	T/T	T/C	Intergenic
**M15-3-2**	chr10	124,110,689	A/A	A/G	Intergenic
**M15-3-2**	chr12	108,257,637	T/T	A/T	Intergenic
**M15-3-2**	chr12	231,274,551	A/A	A/G	Intron
**M15-3-2**	chr12	246,171,974	A/A	A/G	Exon
**M15-3-3**	chr02	168,715,392	C/C	T/C	Intron
**M15-3-3**	chr04	56,996,918	A/A	A/G	Intergenic
**M15-3-3**	chr04	69,708,005	T/T	T/C	Intergenic
**M15-3-3**	chr04	134,242,570	A/A	A/G	Intergenic
**M15-3-3**	chr04	231,091,908	C/C	A/C	Exon
**M15-3-3**	chr04	238,116,670	T/T	T/C	Exon
**M15-3-3**	chr05	2,355,579	T/T	T/C	Exon
**M15-3-3**	chr05	17,488,306	G/G	A/G	Exon
**M15-3-3**	chr05	236,297,933	C/C	A/C	Intergenic
**M15-3-3**	chr07	27,245,627	A/A	A/T	Intron
**M15-3-3**	chr07	236,497,846	C/C	T/C	Exon
**M15-3-3**	chr08	41,635,290	T/T	T/C	Intergenic
**M15-3-3**	chr09	264,042,356	G/G	A/G	Intergenic
**M15-3-3**	chr10	1,440,781	G/G	G/C	Intron
**M15-3-3**	chr11	71,589,514	A/A	A/T	Intergenic
**M15-3-3**	chr12	16,869,260	T/T	T/C	Intergenic
**M15-3-3**	chr12	202,414,918	A/A	A/G	Intron
**M15-3-3**	PGAv.1.6.scaffold959	414,776	C/C	A/C	Exon
**M15-3-3**	PGAv.1.6.scaffold959	414,777	C/C	G/C	Exon
**M15-3-3**	PGAv.1.6.scaffold1076	626,981	G/G	A/G	Exon
**M15-3-3**	PGAv.1.6.scaffold1110	339,895	C/C	A/C	Intron
**M15-3-3**	PGAv.1.6.scaffold1235	323,447	A/A	A/T	Intergenic
**M15-3-3**	PGAv.1.6.scaffold1339	189,070	G/G	A/G	Intergenic
**M15-3-3**	PGAv.1.6.scaffold1463	359,768	T/T	A/T	Intergenic
**M15-3-3**	PGAv.1.6.scaffold2076	17,330	A/A	A/G	Intergenic
**M30-3-1**	chr01	195,596,653	C/C	A/C	Exon
**M30-3-1**	chr03	278,801,137	A/A	A/G	Intergenic
**M30-3-1**	chr05	229,488,328	T/T	A/T	Intergenic
**M30-3-1**	chr06	31,044,111	C/C	A/C	Intergenic
**M30-3-1**	chr06	124,125,756	T/T	T/C	Intergenic
**M30-3-1**	chr06	229,568,810	C/C	T/C	Intron
**M30-3-1**	chr07	250,977,513	C/C	A/C	Intergenic
**M30-3-1**	chr08	135,983,944	G/G	A/G	Exon
**M30-3-1**	PGAv.1.6.scaffold959	414,776	C/C	A/C	Exon
**M30-3-1**	PGAv.1.6.scaffold959	414,777	C/C	G/C	Exon
**M30-3-1**	PGAv.1.6.scaffold1263	397,886	G/G	G/T	Intergenic
**M30-3-2**	chr01	32,100,231	T/T	A/T	Intron
**M30-3-2**	chr01	66,772,164	G/G	A/G	Exon
**M30-3-2**	chr02	8,286,136	G/G	A/G	Intron
**M30-3-2**	chr02	144,294,495	C/C	T/C	Intergenic
**M30-3-2**	chr02	159,953,550	T/T	T/C	Intergenic
**M30-3-2**	chr03	31,870,118	C/C	T/C	Intergenic
**M30-3-2**	chr03	265,851,915	A/A	A/T	Exon
**M30-3-2**	chr03	278,327,993	A/A	A/G	Intergenic
**M30-3-2**	chr04	3,303,984	C/C	A/C	Exon
**M30-3-2**	chr04	174,341,443	T/T	T/C	Intergenic
**M30-3-2**	chr04	239,356,265	G/G	A/G	Exon
**M30-3-2**	chr04	239,489,331	T/T	T/C	Intron
**M30-3-2**	chr05	237,127,962	T/T	T/C	Exon
**M30-3-2**	chr06	240,254,043	C/C	A/C	Exon
**M30-3-2**	chr07	4,233,383	T/T	T/C	Intron

Individual ID	Chromosome ID	Position on chromosome	Genotype in original line	Genotype in mutant line	Feature
**M30-3-2**	chr09	243,773,988	A/A	A/G	Intergenic
**M30-3-2**	chr09	266,732,330	A/A	A/G	Intergenic
**M30-3-2**	chr10	226,444,191	C/C	T/C	Intergenic
**M30-3-2**	chr11	182,359,977	G/G	G/C	Intron
**M30-3-2**	chr12	142,010,366	G/G	G/T	Intron
**M30-3-2**	PGAv.1.6.scaffold504	897,505	A/A	A/T	Intron
**M30-3-2**	PGAv.1.6.scaffold866	1,053,544	G/G	G/T	Exon
**M30-3-2**	PGAv.1.6.scaffold2301	2,779	T/T	T/C	Intergenic
**M30-3-3**	chr03	37,233,803	C/C	G/C	Exon
**M30-3-3**	chr08	68,137,032	A/A	A/G	Intergenic
**M30-3-3**	PGAv.1.6.scaffold959	414,776	C/C	A/C	Exon
**M30-3-3**	PGAv.1.6.scaffold959	414,777	C/C	G/C	Exon
**M30-3-3**	PGAv.1.6.scaffold1077	209,815	G/G	A/G	Intergenic
**M30-3-3**	PGAv.1.6.scaffold21166	95	G/G	A/G	
**M60-3-1**	chr02	116,601,144	C/C	A/C	Intron
**M60-3-1**	chr03	67,895,808	A/A	A/G	Exon
**M60-3-1**	chr04	157,052,358	A/A	A/G	Intergenic
**M60-3-1**	chr05	2,355,579	T/T	T/C	Exon
**M60-3-1**	chr06	3,355,232	A/A	A/C	Intergenic
**M60-3-1**	chr06	234,629,209	A/A	A/G	Exon
**M60-3-1**	chr12	175,118,553	C/C	T/C	Exon
**M60-3-1**	PGAv.1.6.scaffold959	414,776	C/C	A/C	Exon
**M60-3-1**	PGAv.1.6.scaffold21484	435	A/A	A/T	
**M60-3-2**	chr01	65,067,190	T/T	T/C	Intron
**M60-3-2**	chr01	66,480,898	G/G	A/G	Exon
**M60-3-2**	chr01	192,588,736	G/G	A/G	Intron
**M60-3-2**	chr02	37,470,986	A/A	A/T	Exon
**M60-3-2**	chr02	39,489,676	C/C	T/C	Intergenic
**M60-3-2**	chr02	114,671,298	G/G	A/G	Exon
**M60-3-2**	chr02	164,750,646	G/G	A/G	Intergenic
**M60-3-2**	chr03	9,407,891	A/A	A/G	Intron
**M60-3-2**	chr03	33,641,605	G/G	A/G	Exon
**M60-3-2**	chr03	97,678,671	G/G	G/T	Exon
**M60-3-2**	chr03	267,327,571	C/C	T/C	Intron
**M60-3-2**	chr04	209,235,635	G/G	G/T	Intron
**M60-3-2**	chr05	1,098,775	G/G	A/G	Intergenic
**M60-3-2**	chr05	7,858,315	C/C	T/C	Intergenic
**M60-3-2**	chr05	11,418,715	C/C	T/C	Intron
**M60-3-2**	chr06	203,920,742	C/C	A/C	Exon
**M60-3-2**	chr07	237,300,425	G/G	A/G	Intergenic
**M60-3-2**	chr08	125,812,307	C/C	A/C	Exon
**M60-3-2**	chr10	11,381,555	A/A	A/T	Exon
**M60-3-2**	chr10	230,736,078	T/T	T/C	Exon
**M60-3-2**	chr11	2,339,033	A/A	A/G	Exon
**M60-3-2**	chr11	2,339,090	T/T	A/T	Exon
**M60-3-2**	chr11	88,494,423	A/A	A/G	Intergenic
**M60-3-2**	chr11	255,032,054	C/C	T/C	Exon
**M60-3-2**	PGAv.1.6.scaffold289	201,570	C/C	T/C	Exon
**M60-3-2**	PGAv.1.6.scaffold1379	416,946	G/G	G/T	Intergenic
**M60-3-2**	PGAv.1.6.scaffold1529	129,375	G/G	A/G	Intron
**M60-3-3**	chr01	43,477,866	C/C	T/C	Intergenic
**M60-3-3**	chr01	66,480,892	T/T	A/T	Exon
**M60-3-3**	chr01	192,588,736	G/G	A/G	Intron
**M60-3-3**	chr01	207,740,897	G/G	T/T	Intron
**M60-3-3**	chr02	52,878,492	G/G	A/G	Intergenic
**M60-3-3**	chr02	75,690,611	G/G	G/T	Intron
**M60-3-3**	chr02	113,065,164	G/G	A/G	Intergenic
**M60-3-3**	chr02	163,888,470	C/C	T/C	Intron
**M60-3-3**	chr03	6,516,769	C/C	T/C	Intron
**M60-3-3**	chr03	32,886,836	C/C	T/C	Intergenic
**M60-3-3**	chr03	261,915,716	A/A	A/T	Intron

Individual ID	Chromosome ID	Position on chromosome	Genotype in original line	Genotype in mutant line	Feature
**M60-3-3**	chr04	207,937,369	G/G	A/G	Intergenic
**M60-3-3**	chr04	229,178,117	A/A	A/G	Intergenic
**M60-3-3**	chr04	239,643,705	A/A	A/G	Exon
**M60-3-3**	chr05	26,667,436	T/T	A/T	Intron
**M60-3-3**	chr06	3,536,140	C/C	T/C	Exon
**M60-3-3**	chr06	237,277,144	G/G	A/G	Exon
**M60-3-3**	chr07	237,359,684	C/C	T/C	Intergenic
**M60-3-3**	chr08	784,957	G/G	G/C	Intron
**M60-3-3**	chr09	47,706,615	T/T	T/C	Exon
**M60-3-3**	chr10	73,560,588	T/T	T/C	Intergenic
**M60-3-3**	chr10	226,035,100	G/G	G/T	Exon
**M60-3-3**	chr10	229,177,747	G/G	A/G	Intron
**M60-3-3**	chr11	69,336,922	A/A	A/G	Intergenic
**M60-3-3**	chr11	254,292,626	T/T	T/C	Exon
**M60-3-3**	chr12	95,260,285	C/C	T/C	Intergenic
**M60-3-3**	chr12	117,007,288	G/G	A/G	Intergenic
**M60-3-3**	PGAv.1.6.scaffold322	969,263	C/C	T/C	Intron
**M60-3-3**	PGAv.1.6.scaffold959	414,776	C/C	A/C	Exon
**M60-3-3**	PGAv.1.6.scaffold959	414,777	C/C	G/C	Exon
**M60-3-3**	PGAv.1.6.scaffold1077	564,957	G/G	A/A	Intergenic
**M60-3-3**	PGAv.1.6.scaffold1110	383,572	G/G	A/G	Intergenic
**S30-1**	chr01	44,660,989	A/A	A/T	Intron
**S30-1**	chr10	1,309,672	G/G	G/T	Intron
**S30-1**	PGAv.1.6.scaffold1470	66,979	T/T	A/T	Exon
**S30-1**	PGAv.1.6.scaffold1909	107,231	A/A	A/G	Exon
**S30-2**	chr01	58,231,898	C/C	A/C	Intergenic
**S30-2**	chr03	31,616,161	G/G	A/G	Exon
**S30-2**	chr07	2,314,141	T/T	T/C	Exon
**S30-2**	chr11	260,367,960	A/A	A/G	Intergenic
**S30-2**	PGAv.1.6.scaffold1470	66,952	G/G	A/G	Exon
**S30-2**	PGAv.1.6.scaffold16173	240	T/T	T/C	
**S30-2**	PGAv.1.6.scaffold16173	275	C/C	T/C	
**S30-3**	chr03	14,718,644	G/G	G/T	Exon
**S30-3**	chr07	175,241,437	G/G	G/T	Intergenic
**S30-3**	PGAv.1.6.scaffold959	414,776	C/C	A/C	Exon
**S30-3**	PGAv.1.6.scaffold959	414,777	C/C	G/C	Exon
**S30-3**	PGAv.1.6.scaffold2254	11,729	C/C	G/C	Exon
**S30-3**	PGAv.1.6.scaffold2254	11,763	A/A	A/G	Exon
**S30-4**	chr01	141,772,572	T/T	T/C	Intergenic
**S30-4**	chr01	256,293,127	G/G	A/G	Exon
**S30-4**	chr02	152,406,675	G/G	G/T	Intron
**S30-4**	chr03	239,119,536	C/C	T/C	Intron
**S30-4**	chr03	242,026,325	T/T	A/T	Intergenic
**S30-4**	chr04	101,748,488	C/C	T/C	Intergenic
**S30-4**	chr04	224,440,596	T/T	A/T	Intergenic
**S30-4**	chr04	238,651,299	G/G	A/G	Intergenic
**S30-4**	chr05	63,516,877	G/G	A/G	Intron
**S30-4**	chr05	104,521,181	G/G	G/T	Intergenic
**S30-4**	chr06	1,158,449	G/G	A/G	Exon
**S30-4**	chr09	9,143,646	G/G	A/G	Exon
**S30-4**	chr09	35,327,355	G/G	A/G	Intron
**S30-4**	chr09	264,161,935	C/C	A/C	Intergenic
**S30-4**	chr10	156,871,318	C/C	A/C	Intergenic
**S30-4**	PGAv.1.6.scaffold322	844,910	C/C	T/C	Exon
**S30-4**	PGAv.1.6.scaffold588	1,292,489	G/G	A/G	Exon
**S60-1**	PGAv.1.6.scaffold959	414,776	C/C	A/C	Exon
**S60-1**	PGAv.1.6.scaffold959	414,777	C/C	G/C	Exon
**S60-1**	PGAv.1.6.scaffold2779	15,226	G/G	G/T	Intergenic
**S60-2**	chr02	144,639,022	G/G	A/A	Exon
**S60-3**	chr02	154,077,022	G/G	A/G	Exon
**S60-3**	chr03	27,704,950	A/A	A/G	Intergenic

Individual ID	Chromosome ID	Position on chromosome	Genotype in original line	Genotype in mutant line	Feature
**S60-3**	chr05	2,355,579	T/T	T/C	Exon
**S60-3**	chr05	179,384,477	G/G	A/G	Exon
**S60-3**	chr06	228,856,195	T/T	A/T	Exon
**S60-3**	chr06	228,856,247	C/C	A/C	Exon
**S60-3**	chr06	228,856,248	G/G	A/G	Exon
**S60-3**	chr07	78,040,451	G/G	A/G	Intergenic
**S60-3**	chr10	229,255,280	T/T	G/T	Intron
**S60-3**	chr12	119,630,868	C/C	T/C	Intergenic
**S60-3**	PGAv.1.6.scaffold1470	66,979	T/T	A/A	Exon
**S60-3**	PGAv.1.6.scaffold1470	67,010	G/G	C/C	Exon
**S60-4**	chr01	186,739,502	C/C	T/C	Intergenic
**S60-4**	chr09	250,188,656	C/C	T/C	Intergenic
**S60-4**	chr11	87,941,251	G/G	G/T	Intergenic
**S60-4**	PGAv.1.6.scaffold959	414,776	C/C	A/C	Exon
**S60-4**	PGAv.1.6.scaffold959	414,777	C/C	G/C	Exon
**S120-1**	chr11	9,219,155	T/T	T/C	Exon
**S120-2**	chr02	150,076,655	G/G	A/G	Intergenic
**S120-2**	chr03	265,123,018	A/A	A/T	Intron
**S120-2**	chr07	245,626,851	C/C	T/C	Intergenic
**S120-2**	chr07	246,432,874	G/G	A/G	Intron
**S120-3**	chr05	96,700,270	A/A	A/G	Intron
**S120-3**	chr07	251,059,198	T/T	T/C	Exon
**S120-3**	chr08	2,838,705	C/C	T/C	Intergenic
**S120-4**	chr07	248,687,759	G/G	G/T	Intron
**S120-4**	chr07	251,059,198	T/T	T/C	Exon

Table S6. Information of small InDels detected by the genotype-by-sequencing (GBS) analysis performed for analysis of mutation frequency

Individual ID	Chromosome Id	Position on chromosome	Genotype in mutant line	Feature
**F15-3-1**	chr05	117760522	-2AA/*	Exon
**F15-3-1**	chr07	2463315	-1T/*	Intron
**F15-3-2**	chr09	219968243	*/-1A	Intergenic
**F15-3-2**	chr12	248828044	*/+1A	Intergenic
**F15-3-3**	chr05	117760522	*/-2AA	Exon
**F15-3-3**	chr11	10825274	*/-1G	Intergenic
**F30-2-1**	chr01	253548821	*/-1T	Intron
**F30-2-1**	chr05	11424255	*/+1G	Intergenic
**F30-2-1**	chr05	117760522	-2AA/*	Exon
**F30-2-2**	chr05	117760522	*/-2AA	Exon
**F30-2-2**	chr07	202123203	-1T/*	Intergenic
**F30-2-3**	chr07	104544413	*/-1A	Intron
**F30-3-1**	chr05	117760522	-2AA/*	Exon
**F30-3-1**	chr08	140333073	*/-1C	Intergenic
**F30-3-1**	PGAv.1.6.scaffold2733	3243	*/-1T	
**F30-3-2**	chr05	117760522	*/-2AA	Exon
**F30-3-3**	chr05	117760522	-2AA/*	Exon
**F30-3-3**	PGAv.1.6.scaffold2189	33587	*/-1G	Intergenic
**F60-1-1**	chr03	10803233	*/-1A	Intergenic
**F60-1-1**	chr05	117760522	*/-2AA	Exon
**F60-1-2**	chr05	117760522	-2AA/*	Exon
**F60-1-2**	chr10	185234138	*/-1A	Intergenic
**F60-1-3**	chr07	235532161	*/-1C	Intron
**F60-2-1**	chr04	693798	*/-1A	Exon
**F60-2-1**	chr04	74799733	*/-1A	Intergenic
**F60-2-1**	chr04	191164692	*/-1T	Intergenic
**F60-2-1**	chr04	218341524	*/+1C	Exon
**F60-2-2**	chr01	49258986	*/-1T	Intergenic
**F60-2-2**	chr01	60766728	*/+1T	Intergenic
**F60-2-2**	chr08	140333073	*/-1C	Intergenic
**F60-2-3**	chr03	8074365	*/+1T	Intron
**F60-2-3**	chr05	117760522	-2AA/*	Exon
**F60-2-3**	chr12	18172191	*/-1A	Exon
**F60-2-3**	chr12	119630839	*/+1T	Intergenic
**F60-3-1**	chr05	117760522	*/-2AA	Exon
**F60-3-2**	PGAv.1.6.scaffold1263	237429	*/+1T	Intergenic
**F60-3-3**	chr06	23117769	*/-1G	Exon

Individual ID	Chromosome Id	Position on chromosome	Genotype in mutant line	Feature
**M15-3-1**	chr03	21410325	*/-1T	Intron
**M15-3-1**	chr03	252183238	*/-1T	Intron
**M15-3-1**	chr05	117760522	-2AA/*	Exon
**M15-3-1**	chr07	181619934	*/+1A	Intergenic
**M15-3-2**	chr05	117760522	*/-2AA	Exon
**M15-3-2**	chr07	220492826	*/+1T	Intergenic
**M15-3-3**	chr09	222132492	-1C	Intergenic
**M30-3-1**	chr05	117760522	*/-2AA	Exon
**M30-3-2**	chr05	117760522	*/-2AA	Exon
**M30-3-2**	chr06	221198064	*/-1G	Intergenic
**M30-3-3**	chr05	34781882	*/-1A	Intron
**M30-3-3**	chr10	229161595	*/-1A	Intron
**M30-3-3**	chr12	248828044	*/+1A	Intergenic
**M60-3-1**	chr05	117760522	*/-2AA	Exon
**M60-3-2**	chr05	117760522	-2AA/*	Exon
**M60-3-3**	chr01	198766072	-1T/*	Intron
**M60-3-3**	chr05	117760522	*/-2AA	Exon
**S30-1**	chr05	117760522	*/-2AA	Exon
**S30-1**	PGAv.1.6.scaffold2162	4282	+1G/*	
**S30-2**	chr01	118537813	*/-2CT	Intergenic
**S30-4**	PGAv.1.6.scaffold959	620628	*/-1T	Exon
**S60-3**	chr05	117760522	*/-2AA	Exon
**S60-3**	chr12	116642991	*/-1C	Intergenic
**S60-4**	chr05	117760522	*/-2AA	Exon
**S60-4**	chr06	201138381	*/-1A	Intergenic
**S120-2**	chr03	15892926	*/-1G	Exon
**S120-2**	chr06	208991839	*/-1T	Intergenic

Table S7. Results of pair-wise *t*-tests between groups of progeny derived from gametophytes irradiated under different conditions

	Pistil-15 Gy-stage 3	Pistil-30 Gy-stage 2	Pistil-30 Gy-stage 3	Pistil-60 Gy-stage 1	Pistil-60 Gy-stage 2	Pistil-60 Gy-stage 3	Stamen-15 Gy-stage 3	Stamen-30 Gy-stage 3	Stamen-60 Gy-stage 3	Seed-30 Gy	Seed-60 Gy	Seed-120 Gy
Pistil-15 Gy-stage 3	-											
Pistil-30 Gy-stage 2	-	-			*[z]							
Pistil-30 Gy-stage 3	-	-	-									
Pistil-60 Gy-stage 1	-	-	-	-								
Pistil-60 Gy-stage 2	-	-	-	-	-						*	*
Pistil-60 Gy-stage 3	-	-	-	-	-	-						
Stamen-15 Gy-stage 3	-	-	-	-	-	-	-				*	**
Stamen-30 Gy-stage 3	-	-	-	-	-	-	-	-				
Stamen-60 Gy-stage 3	-	-	-	-	-	-	-	-	-			
Seed-30 Gy	-	-	-	-	-	-	-	-	-	-		
Seed-60 Gy	-	-	-	-	-	-	-	-	-	-	-	
Seed-120 Gy	-	-	-	-	-	-	-	-	-	-	-	-

[z] Differences detected at *P*-value < 0.05 and *P*-value < 0.01 are indicated by one and two asterisks, respectively.

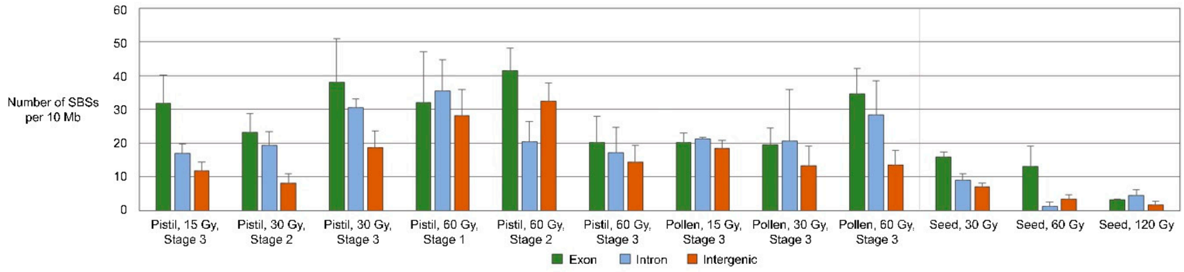

Figure S1. Frequency of single-base substitutions (SBSs) according to location on genome in groups of progeny derived from gametophytes irradiated under different conditions
